# *Plasmopara viticola* infection affects mineral elements allocation and distribution in *Vitis vinifera* leaves

**DOI:** 10.1038/s41598-020-75990-x

**Published:** 2020-10-30

**Authors:** Stefano Cesco, Anna Tolotti, Stefano Nadalini, Stefano Rizzi, Fabio Valentinuzzi, Tanja Mimmo, Carlo Porfido, Ignazio Allegretta, Oscar Giovannini, Michele Perazzolli, Guido Cipriani, Roberto Terzano, Ilaria Pertot, Youry Pii

**Affiliations:** 1 Faculty of Science and Technology, Free University of Bozen-Bolzano, Piazza Università 5, 39100 Bolzano, Italy; 2 Department of Agricultural, Food, Environmental and Animal Sciences, University of Udine, 33100 Udine, Italy; 3 Competence Centre for Plant Health, Free University of Bozen-Bolzano, 39100 Bolzano, Italy; 4 Department of Soil, Plant and Food Sciences, University of Bari “Aldo Moro”, 70126 Bari, Italy; 5 Department of Sustainable Agro-Ecosystems and Bioresources, Research and Innovation Centre, Fondazione Edmund Mach, 38010 San Michele all’Adige, Italy; 6 Center Agriculture Food Environment (C3A), University of Trento, 38010 San Michele all’Adige, Italy

**Keywords:** Biotic, Plant physiology

## Abstract

*Plasmopara viticola* is one of the most important pathogens infecting *Vitis vinifera* plants. The interactions among *P. viticola* and both susceptible and resistant grapevine plants have been extensively characterised, at transcriptomic, proteomic and metabolomic levels. However, the involvement of plants ionome in the response against the pathogen has been completely neglected so far. Therefore, this study was aimed at investigating the possible role of leaf ionomic modulation during compatible and incompatible interactions between *P. viticola* and grapevine plants. In susceptible cultivars, a dramatic redistribution of mineral elements has been observed, thus uncovering a possible role for mineral nutrients in the response against pathogens. On the contrary, the resistant cultivars did not present substantial rearrangement of mineral elements at leaf level, except for manganese (Mn) and iron (Fe). This might demonstrate that, resistant cultivars, albeit expressing the resistance gene, still exploit a pathogen response mechanism based on the local increase in the concentration of microelements, which are involved in the synthesis of secondary metabolites and reactive oxygen species. Moreover, these data also highlight the link between the mineral nutrition and plants’ response to pathogens, further stressing that appropriate fertilization strategies can be fundamental for the expression of response mechanisms against pathogens.

## Introduction

Grapevine is one of the most important crops worldwide, for production of fresh fruits, raisins, juices and wine^1^ and, within the genus *Vitis*, *V. vinifera* L. is the most relevant species for the wine industry. In 2018, the total surface cultivated with *V. vinifera* and the overall wine production was approximatively 7.5 million Ha and 30,000 million litres, respectively (https://www.oiv.int/). However, grapevine can be affected by a wide number diseases caused by various pathogenic organisms, which can cause production losses both in terms of quantity and quality with considerable negative impacts on wine industry^2^. *Plasmopara viticola* is one of the most important phytopathogens of *V. vinifera*, together with *Botrytis cinerea* and *Erysiphe necator*^3^. Downey mildew symptoms are characterised by initial yellow and oily spots on leaves and other green parts of the plant that evolve in necrosis and death of infected tissues, including bunches, possibly resulting in a total crop loss^4^. In order to control the disease, several fungicide treatments, including the copper (Cu)-based ones, are commonly applied in vineyards^3,5,6^. However, there are growing concerns about the possible negative impact of synthetic chemical fungicides and Cu on human health and the environment^7–9^. In particular, Cu can accumulate in the surface horizon of the soil and have adverse effects on soil biota^10–12^, as well as, on plants^7,13–18^. Therefore, with the aim of reducing the impact of synthetic chemical and Cu-based fungicides, the implementation of more sustainable practices aimed at controlling phytopathogen in viticulture are highly encouraged^7^.

Despite the high importance of downy mildew on grapevine production, the molecular bases of *P. viticola* pathogenesis are still largely unknown^2^. Nonetheless, several studies have highlighted that, during the early stages of infection (i.e. hyphal structures and haustoria development), *P. viticola* expresses genes involved in the ATP synthesis and in the active acquisition of mineral elements from the host^19,20^. On the other hand, grapevine plant responses to *P. viticola* have been thoroughly characterised at transcriptomic, proteomic and metabolomic levels^21–26^. In compatible interactions between the host and the pathogen, grapevine plants up-regulate genes related to the secondary metabolism, defence and response to external stimuli, whilst genes involved in the photosynthesis and the carbon metabolism are mostly down-regulated^25^. Transcriptional modulations were detected in micro-dissected stomata (i.e. the primary site of *P. viticola* infection) and surroundings cells, suggesting that grapevine plants can react to the pathogen by eliciting both a site-specific response and a short distance signal(s) from the stomata to neighbouring cells^27^. In contrast, incompatible interactions are characterised by the up-regulation of stress and defence-related genes, as for instance the Pathogenesis Related (PR) proteins^3,28^. Several Quantitative Trait Loci (QTLs), named *Resistance to P. viticola* (*Rpv*) genes, have been identified in different grapevine species and they have been associated with major phenotypic traits of resistance to downy mildew disease. In particular, *Rpv1* and *Rpv2* were found to be responsible for the resistance in *Muscadinia rotundifolia*^29^, *Rpv3* was associated with the resistance in ‘Villard blanc’^30^, whilst the resistance traits in *V. amurensis* was ascribed to *Rpv8*,* Rpv10* and *Rpv12*^31–33^. Since the discovery of the resistance traits to *P. viticola*, breeding programs have been undertaken in order to introduce the features related to downy mildew resistance in susceptible species^34–38^. Thus, the use of grapevine genotypes resistant to *P. viticola* infection might represent an important tool to improve the sustainability of viticulture^39^, in particular considering the current restriction on the use of Cu in the European Union^40^. Depending on the specific QTL and the grapevine genotype, the resistance mechanisms elicited by the *Rpv* genes can involve different responses, as for instance the accumulation of callose and lignin^41–43^, the hypersensitive response^33,44^, synthesis of phytoalexins^45,46^, the accumulation of phenolics in the infected tissues^47,48^, the induction of either cell necrosis^38,44,49^ or peroxidase activity^50,51^.

Recent studies have highlighted that also the ionome profile of plants might represent an important factor determining the success of the infection process by a pathogenic organism^52,53^. Mineral elements, both macro- and microelements, play a fundamental role in plants, being essential for the life cycles completion. They are involved in a plethora of cell functions, including primary and secondary metabolisms, energy production, defence, signals transduction, genes regulation, hormones perception, reproduction, enzyme functioning, as well as in maintaining cell structure^54^. In addition, an adequate intracellular concentration of essential metal ions was recognised as a necessary prerequisite for both pathogen virulence and plant defence responses^55,56^. In particular, considering the role played by metal ions in living organisms, it is conceivable that intracellular mineral concentrations of essential mineral elements can have a strong impact on a wide range of pathogens and on their ability to establish an interaction with host plants^57^. A recent research carried out on olive trees infected with the bacterial pathogen *Xylella fastidiosa* demonstrated that the resistant variety “Leccino”, which usually shows low or no symptoms, was characterised by a different ionomic signature as compared to the symptomatic susceptible variety (e.g. “Ogliarola salentina”)^53^. In particular, increases in calcium (Ca) and manganese (Mn) leaf concentration were detected, suggesting that these mineral elements might be related to the enhanced resistance of the “Leccino” variety against *X. fastidiosa*^53^. Similarly, it was reported that the ionomic balance in *Lactuca sativa* L. infected with the bacterium *Xanthomonas campestris* pv. *vitians* correlated with the degree of resistance showed by different lettuce varieties^52^.

Starting from this knowledge background, we investigated whether the resistance mechanisms showed by resistant grapevine cultivars, beside the specific activities of *Rpv* genes, might be also associated to the modulation of the nutrient balance in the plants. The ionomic signature of a plant tissue is the result of the regulation of transporter-based fluxes of mineral elements within the plant and among the different tissues, playing the leaf a relevant role^58^. The present study was undertaken on two *Plasmopara*-sensitive grapevine cultivars, Cabernet Sauvignon and Sauvignon Blanc, widely cultivated in wine-producing Countries (approx. 340 thousand and 120 thousand Ha, respectively) (https://www.oiv.int/public/medias/5888/en-distribution-of-the-worlds-grapevine-varieties.pdf), and the respective resistant cultivars (Sauvignon Kretos and Cabernet Volos), bearing the *Rpv12* gene. After the inoculation with *P. viticola*, the ionomic signature of leaves was assessed using standard (e.g. Inductively Coupled Plasma-Optical Emission Spectroscopy) and chemical imaging (e.g. micro X-Ray Fluorescence spectroscopy) techniques, to unravel whether the concentration and/or the distribution of mineral elements could play a role in the response to the pathogen. To elucidate the mechanisms controlling the mineral element allocation and re-distribution upon *P. viticola* infection, the expression of selected genes, encoding mineral element transporters, was also assessed in leaf tissues.

## Results

### Mineral element distribution in *Plasmopara viticola* inoculated leaves

**Figure 1:**
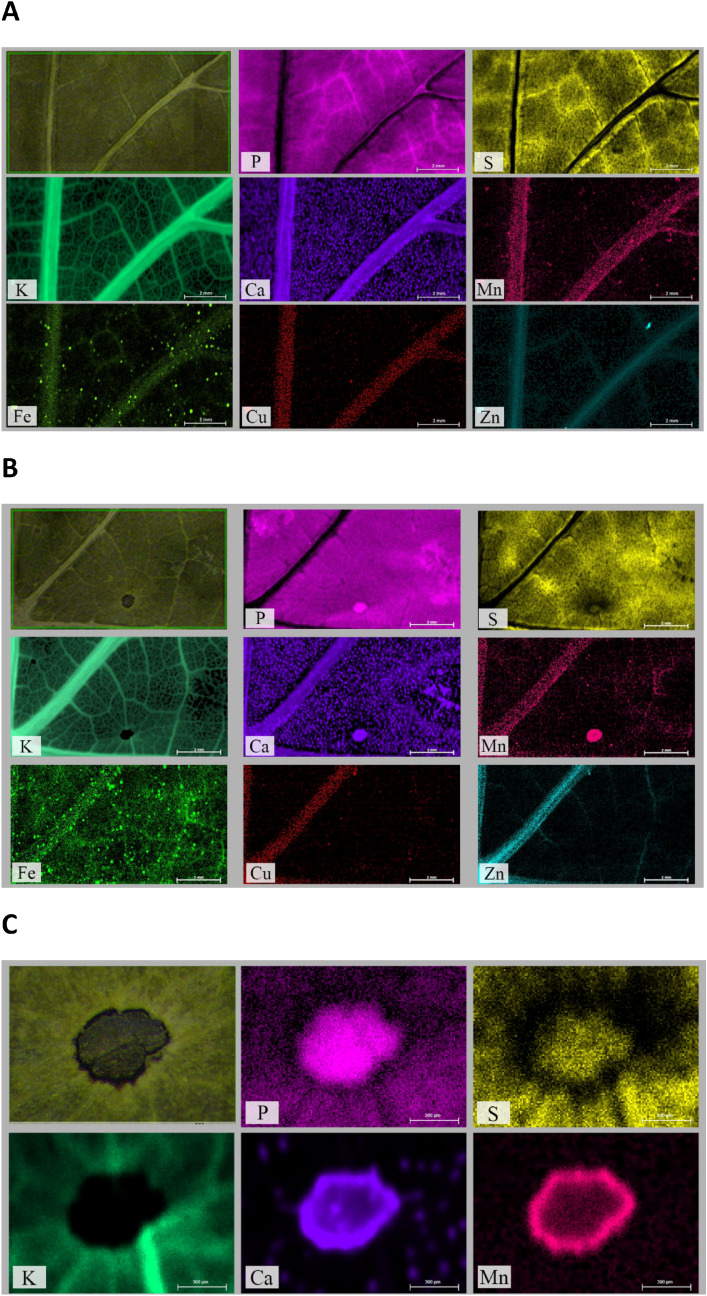
Micro-focused X-ray fluorescence (μXRF) analyses of Sauvignon Blanc leaves. (**A**) Representative μ-XRF distribution maps for Sauvignon Blanc leaves. (**B**) Representative μ-XRF distribution maps for Sauvignon Blanc leaves inoculated with *Plasmopara viticola*. (**C**) Close-up of μ-XRF distribution maps for Sauvignon Blanc leaves inoculated with *P. viticola* at level of the infection spot shown in (**B**). For each mineral element specified by the abbreviation at the bottom of each panel, brighter colours correspond to higher mineral element concentrations.

Six days after *P. viticola* inoculation, leaves of grapevine plants belonging to susceptible cultivars (Sauvignon Blanc and Cabernet Sauvignon) presented the typical symptoms of downy mildew, whilst resistant cultivars (Sauvignon Kretos and Cabernet Volos) did not (data not shown). Leaves of susceptible cultivars were subjected to µXRF analysis in order to unravel mineral element distribution as affected by the host–pathogen interaction. Figure 1A presents the typical mineral element distribution in a representative portion of grapevine leaves (approximately located near the petiole sinus including the midrib and nearby primary vein) belonging to the non-inoculated Sauvignon Blanc. Figure 1B reports the distribution maps of mineral elements in an inoculated Sauvignon Blanc leaf, recorded approximately in the same region as for the leaf of Fig. 1A. Although the spatial allocation of mineral elements in the grapevine mesophyll is comparable between inoculated and non-inoculated samples, several mineral elements revealed an abnormal distribution at the level of infected spots (Fig. 1B). Infected spots were completely devoid of potassium (K), whilst phosphorus (P), Ca and Mn seemed to be accumulated within the spots (Fig. 1B). When examined at higher magnification (Fig. 1C), it was possible to confirm the almost complete depletion of K and an accumulation of both P and Ca in the infected spot. In addition, Mn and Ca showed an accumulation in correspondence of the spot borders, whereas sulphur (S) displayed a preferential allocation at the centre of the infected spot (Fig. 1C). Iron (Fe) homogeneously increased in the tissue of inoculated leaves (Fig. 1B) compared to non-inoculated leaves (Fig. 1A). Zinc (Zn) appeared more concentrated in the primary veins and copper (Cu) did not show any variation in the allocation in inoculated as compared to non-inoculated tissues (Fig. 1A,B). The distribution maps of mineral elements obtained for leaves of Cabernet Sauvignon were substantially congruent to those described above, whilst no alteration in the distribution maps of mineral elements was highlighted in the inoculated Sauvignon Kretos and Cabernet Volos as compared to the non-inoculated samples (data not shown).

### Ionomic signatures of *Plasmopara viticola* inoculated leaves

**Figure 2:**
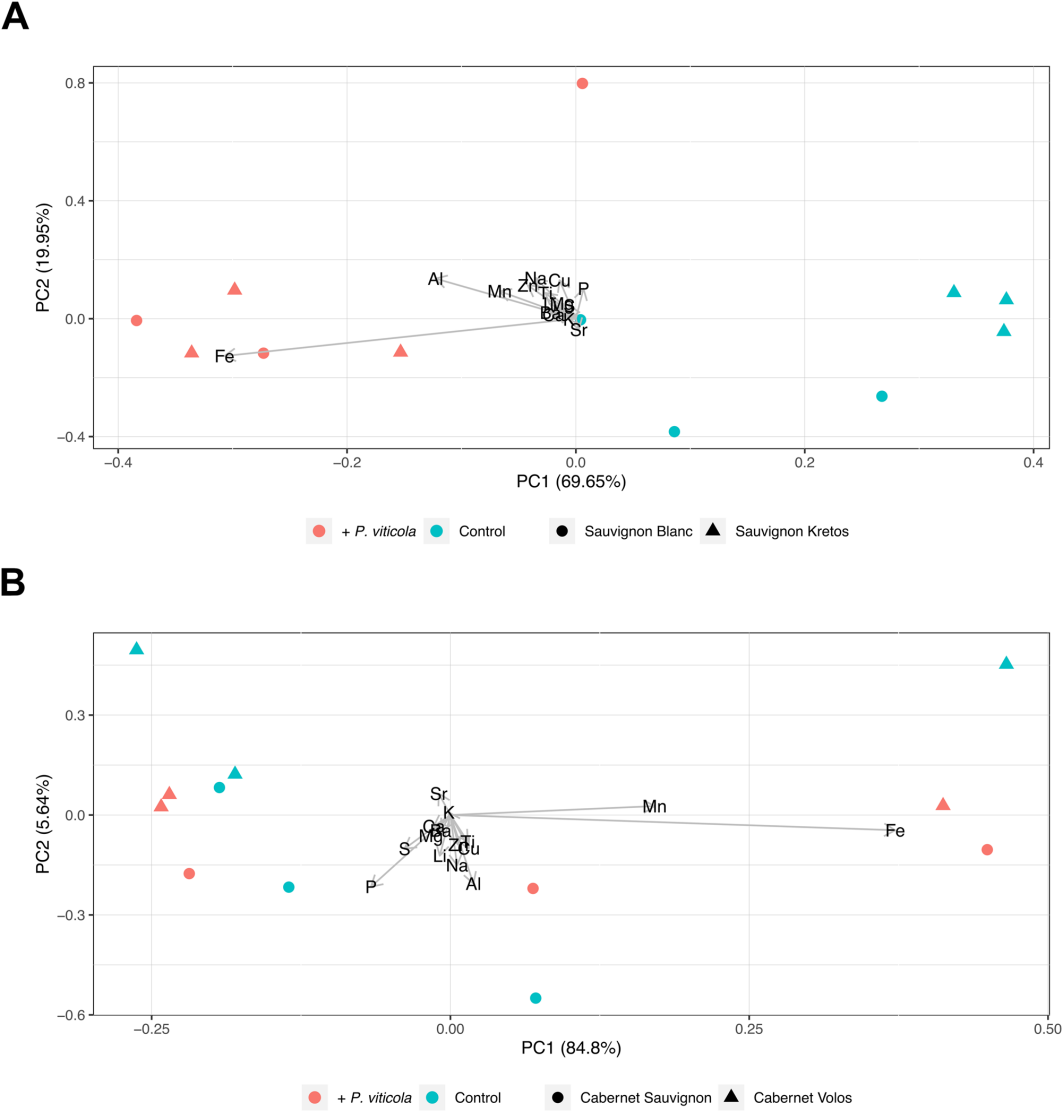
Principal Component Analyses of ionomic datasets. (**A**) Scatterplot representing the modification of leaves ionome in Sauvignon Blanc and Sauvignon Kretos both non-inoculated and inoculated with *Plasmopara viticola*. (**B**) Scatterplot representing the modification of leaves ionome in*.* Cabernet Sauvignon and Cabernet Volos both non-inoculated and inoculated with *Plasmopara viticola*. and by the infection with *P. viticola*.

To further investigate whether the alterations in the distribution maps of mineral elements were due either to a differential allocation of mineral elements or to an increase in the uptake and/or translocation of minerals, leaves ionomic profiling was undertaken using the ICP-OES technique. The Principal Component Analysis (PCA) carried out on the whole dataset (i.e. white and red cultivars, susceptible and resistant, inoculated and non-inoculated with *P. viticola*) generated a two components model explaining 86.88% of the total variance with no clustering of samples (Supplementary Figure 1). On the other hand, the PCA of white cultivars generated a two components model accounting for 89.60% of the total variance, showing a clear separation between inoculated and non-inoculated samples along the firs principal component 1 (PC1) (Fig. 2A). In addition, within the non-inoculated samples also the separation between Sauvignon Blanc and Sauvignon Kretos was highlighted (Fig. 2A). The mineral elements determining the separation along PC1 were mainly Fe and Mn (Fig. 2A), as found in the mineral element distribution maps (Fig. 1). Conversely, the PCA of the red varieties explained 90.44% of the total variance, with no clear clustering of samples (Fig. 2B).

**Figure 3:**
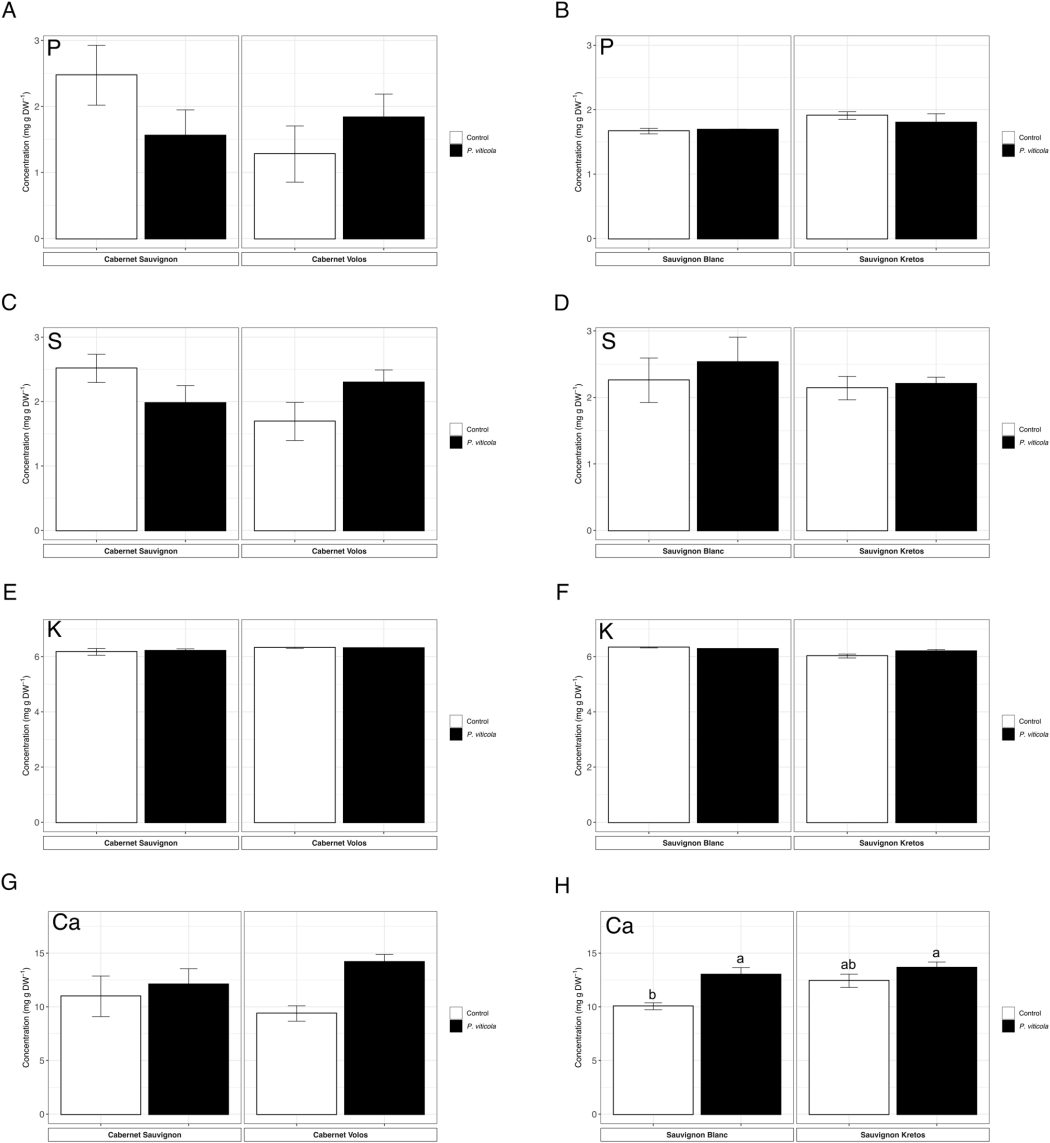
Macroelement concentration. (**A**) Phosphorus concentration in Cabernet Sauvignon and Cabernet Volos, both non-inoculated and inoculated with *Plasmopara viticola*. (**B**) Phosphorus concentration in Sauvignon Blanc and Sauvignon Kretos, both non-inoculated and inoculated with *P. viticola*. (**C**) Sulphur concentration in Cabernet Sauvignon and Cabernet Volos, both non-inoculated and inoculated with *P. viticola*. (**D**) Sulphur concentration in Sauvignon Blanc and Sauvignon Kretos, both non-inoculated and inoculated with *P. viticola*. (**E**) Potassium concentration in Cabernet Sauvignon and Cabernet Volos, both non-inoculated and inoculated with *P. viticola*. (**F**) Potassium concentration in Sauvignon Blanc and Sauvignon Kretos, both non-inoculated and inoculated with *P. viticola*. (**G**) Calcium concentration in Cabernet Sauvignon and Cabernet Volos, both non-inoculated and inoculated with *P. viticola*. (**H**) Calcium concentration in Sauvignon Blanc and Sauvignon Kretos, both non-inoculated and inoculated with *P. viticola*. The data are reported as means ± SE of three biological replicates. Different letters indicate significant differences according to one-way ANOVA with Tukey post hoc tests (*p* < 0.05). Letters were omitted when no significant differences were found.

In spite of the visible alteration observed in the distribution maps of P, the ionomic analyses revealed no differences in the total concentration of P in the leaves of both cultivars, independently from the genotype and *P. viticola* inoculation (Fig. 3A,B). The same also applied for S (Fig. 3C,D), K (Fig. 3E,F) and Ca, at least in the red cultivars (Fig. 3G). Interestingly, the concentration of Ca in the leaves of Sauvignon Blanc was increased by the inoculation with *P. viticola* (Fig. 3H).

**Figure 4:**
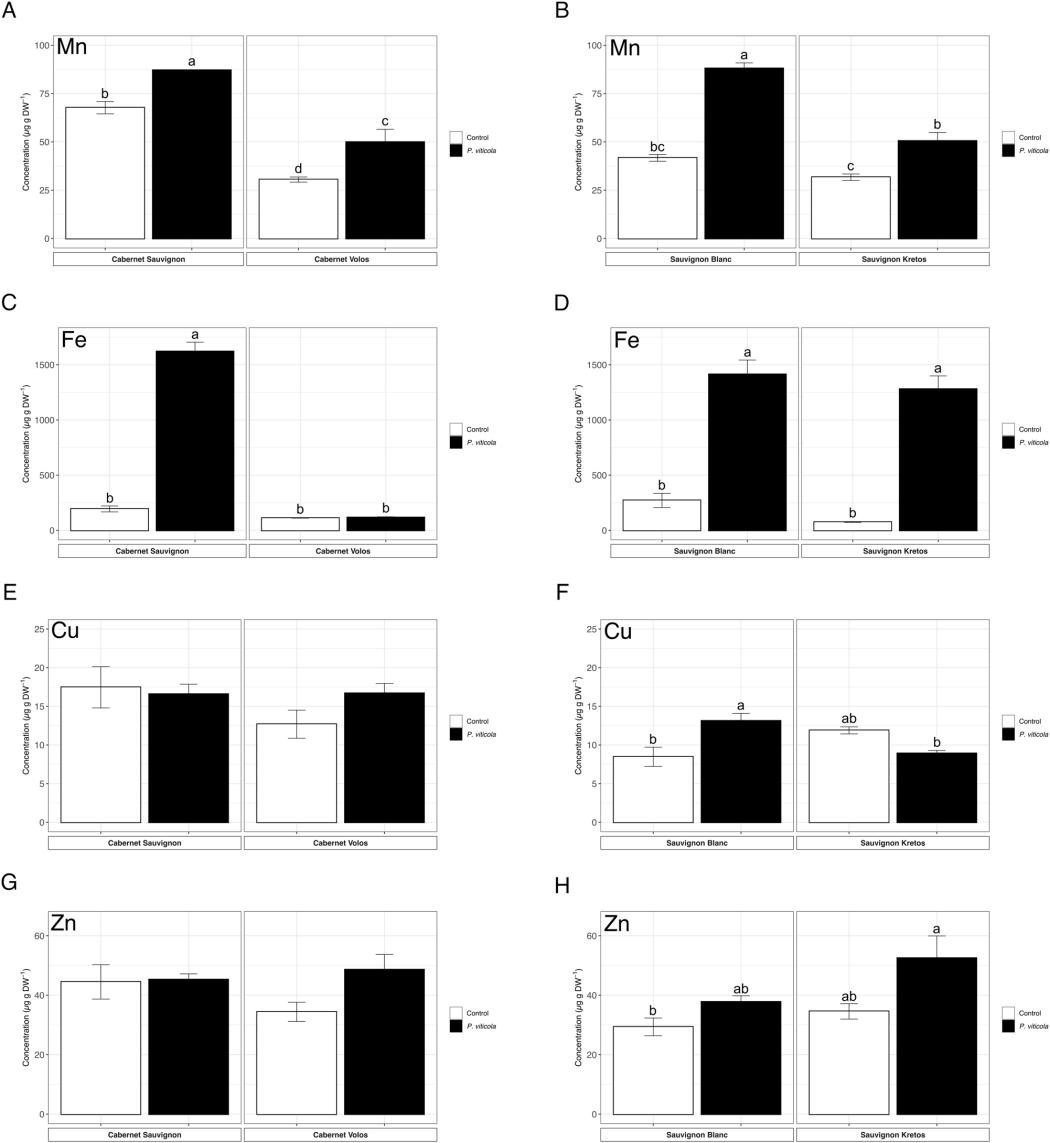
Microelement concentration. (**A**) Manganese concentration in Cabernet Sauvignon and Cabernet Volos, both non-inoculated and inoculated with *Plasmopara viticola*. (**B**) Manganese concentration in Sauvignon Blanc and Sauvignon Kretos, both non-inoculated and inoculated with *P. viticola*. (**C**) Iron concentration in Cabernet Sauvignon and Cabernet Volos, both non-inoculated and inoculated with *P. viticola*. (**D**) Iron concentration in Sauvignon Blanc and Sauvignon Kretos, both non-inoculated and inoculated with *P. viticola*. (**E**) Copper concentration in Cabernet Sauvignon and Cabernet Volos, both non-inoculated and inoculated with *P. viticola*. (**F**) Copper concentration in Sauvignon Blanc and Sauvignon Kretos, both non-inoculated and inoculated with *P. viticola*. (**G**) Zinc concentration in Cabernet Sauvignon and Cabernet Volos, both non-inoculated and inoculated with *P. viticola*. (**H**) Zinc concentration in Sauvignon Blanc and Sauvignon Kretos, both non-inoculated and inoculated with *P. viticola*. The data are reported as means ± SE of three biological replicates. Different letters indicate significant differences according to one-way ANOVA with Tukey post hoc tests (*p* < 0.05). Letters were omitted when no significant differences were found.

The Mn concentration increased in Cabernet Sauvignon and Sauvignon Blanc leaves inoculated with *P. viticola* as compared to non-inoculated plants (Fig. 4A,B), in agreement with the Mn distribution maps. Similarly, a higher concentration of Mn was also found in *P. viticola*-inoculated Cabernet Volos and Sauvignon Kretos by about 60% as compared to non-inoculated leaves (Fig. 4A,B). The Fe concentration was increased by about 8 and 5 times in *P. viticola*-inoculated Cabernet Sauvignon and Sauvignon Blanc as compared to non-inoculated samples, respectively (Fig. 4C,D). Consistently, a 16-fold increase in the Fe concentration was also detected in the *P. viticola*-inoculated leaves of Sauvignon Kretos with respect to non-inoculated Sauvignon Kretos leaves (Fig. 4D), whilst no alterations were detected in Cabernet Volos leaves (Fig. 4C).

Concerning Cu, red and white cultivars showed a discrepancy in the behaviour (Fig. 4E,F). In both the Cabernet Sauvignon and Cabernet Volos, the *P. viticola* inoculation did not cause any variation in the Cu concentration as compared to the respective non-inoculates leaves (Fig. 4E). The Cu concentration was enhanced by about 70% in the inoculated Sauvignon Blanc leaves and reduced by about 30% in the inoculated Sauvignon Kretos leaves as compared to the respective non-inoculated leaves (Fig. 4F). Similarly, Zn concentration was not affected by *P. viticola* inoculation in Cabernet Sauvignon and in Cabernet Volos (Fig. 4G), whereas it was increased by 30% and 50% in Sauvignon Blanc and Sauvignon Kretos as compared to the non-inoculated leaves, respectively (Fig. 4H).

### Gene expression analyses in *Plasmopara viticola* inoculated leaves

#### Sulphate transporters

On the bases of the phylogenetic analysis, five sulphate transporters belonging to the group 3 (i.e.* VvSultr3.1*,* VvSultr3.2*,* VvSultr3.3*,* VvSultr3.4*,* VvSultr3.5*) were identified in *V. vinifera* genome. However, the relative mRNA levels of *VvSultr3.4* resulted not detectable in our experimental conditions.

**Figure 5:**
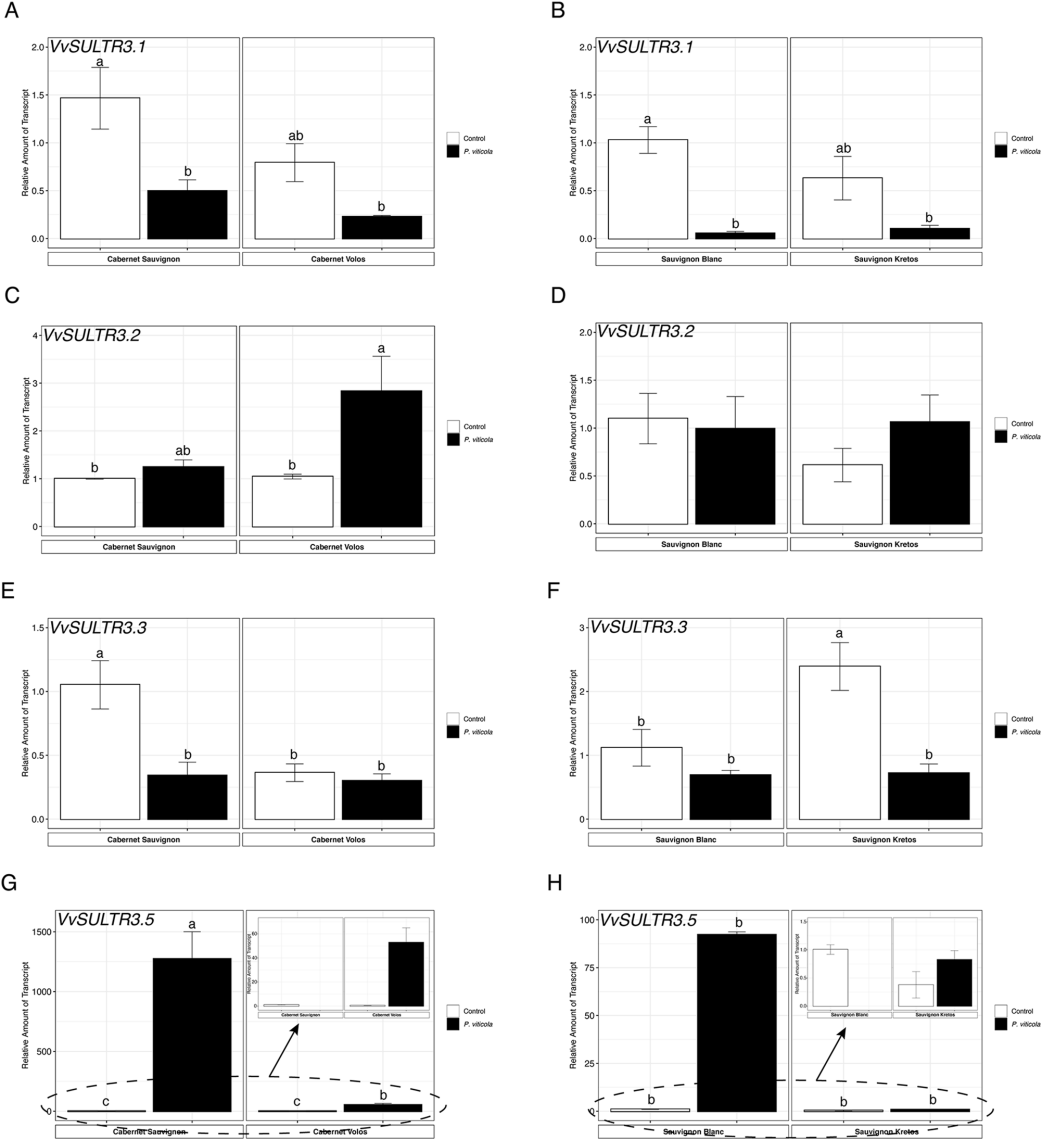
Quantitative RT-PCR analyses of selected sulphate transport genes (*VvSULTR*) in grapevine leaves. (**A**) Assessment of *VvSULTR3.1* gene expression in Cabernet Sauvignon and Cabernet Volos, both non-inoculated and inoculated with *Plasmopara viticola*. (**B**) Assessment of *VvSULTR3.1* gene expression in Sauvignon Blanc and Sauvignon Kretos, both non-inoculated and inoculated with *P. viticola*. (**C**) Assessment of *VvSULTR3.2* gene expression in Cabernet Sauvignon and Cabernet Volos, both non-inoculated and inoculated with *P. viticola*. (**D**) Assessment of *VvSULTR3.2* gene expression in Sauvignon Blanc and Sauvignon Kretos, both non-inoculated and inoculated with *P. viticola*. (**E**) Assessment of *VvSULTR3.3* gene expression in Cabernet Sauvignon and Cabernet Volos, both non-inoculated and inoculated with *P. viticola*. (**F**) Assessment of *VvSULTR3.3* gene expression in Sauvignon Blanc and Sauvignon Kretos, both non-inoculated and inoculated with *P. viticola*. (**G**) Assessment of *VvSULTR3.5* gene expression in Cabernet Sauvignon and Cabernet Volos, both non-inoculated and inoculated with *P. viticola*. (**H**) Assessment of *VvSULTR3.5* gene expression in Sauvignon Blanc and Sauvignon Kretos, both non-inoculated and inoculated with *P. viticola*. The data were normalised to the *tubulin* and *EF1α* housekeeping genes. The relative expression ratios were calculated using untreated parental genotypes as a calibrator sample. The values reported are means ± SE of three biological replicates. Different letters indicate significant differences according to one-way ANOVA with Tukey post hoc tests (*p* < 0.05).

The expression of *VvSULTR3.1* was down-regulated in inoculated as compared to non-inoculated leaves of the susceptible cultivars and it showed a down-regulation trend in the inoculated resistant cultivars, albeit not significantly (Fig. 5A,B). *VvSULTR3.2* was up-regulated in inoculated Cabernet Volos leaves and it was not modulated in the other samples analysed (Fig. 5C,D). The expression of *VvSULTR3.3* had a different behaviour, depending on the cultivar (Fig. 5E,F). In the red cultivars, *P. viticola* inoculation caused a down-regulation of *VvSULTR3.3* only in the susceptible one, whilst it was not modulated in Cabernet Volos (Fig. 5E). In the white cultivars, *P. viticola* inoculation decreased the expression of *VvSULTR3.3* in Sauvignon Kretos leaves and not in Sauvignon Blanc compared to the respective non-inoculated leaves (Fig. 5F).

The expression of the *VvSULTR3.5* was strongly up-regulated in inoculated as compared to non-inoculated susceptible cultivars, by about 1500 and 100-fold in the red and white cultivar, respectively (Fig. 5G,H). The pathogen inoculation up-regulated *VvSULTR3.5* in Cabernet Volos leaves as compared to the non-inoculated plants (Fig. 5G).

#### Iron transporters

**Figure 6:**
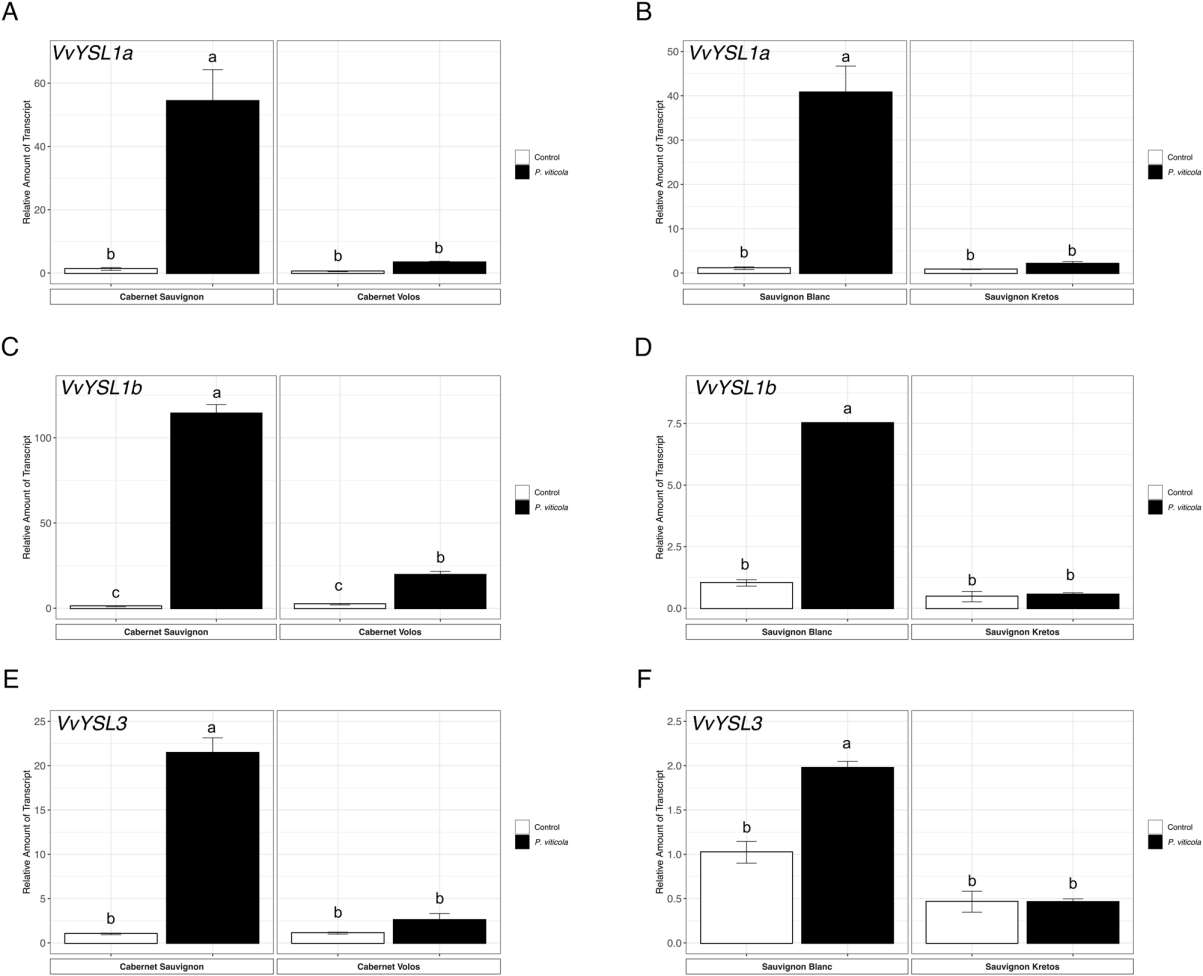
Quantitative RT-PCR analyses of genes possibly involved the Fe partitioning (*VvYSL1a*,* VvYSL1b and VvYSL3*) in grapevine leaves. (**A**) Assessment of *VvYSL1a* gene expression in Cabernet Sauvignon and Cabernet Volos, both non-inoculated and inoculated with *P. viticola*. (**B**) Assessment of *VvYSL1a* gene expression in Sauvignon Blanc and Sauvignon Kretos, both non-inoculated and inoculated with *P. viticola*. (**C**) Assessment of *VvYSL1b* gene expression in Cabernet Sauvignon and Cabernet Volos, both non-inoculated and inoculated with *P. viticola*. (**D** )Assessment of *VvYSL1b* gene expression in Sauvignon Blanc and Sauvignon Kretos, both non-inoculated and inoculated with *P. viticola*. (**E**) Assessment of *VvYSL3* gene expression in Cabernet Sauvignon and Cabernet Volos, both non-inoculated and inoculated with *P. viticola*. (**F**) Assessment of *VvYSL3* gene expression in Sauvignon Blanc and Sauvignon Kretos, both non-inoculated and inoculated with *P. viticola*. The data were normalised to the *tubulin* and *EF1α* housekeeping gene. The relative expression ratios were calculated using untreated parental genotypes as a calibrator sample. The values reported are means ± SE of three biological replicates. Different letters indicate significant differences according to one-way ANOVA with Tukey post hoc tests (*p* < 0.05).

**Figure 7:**
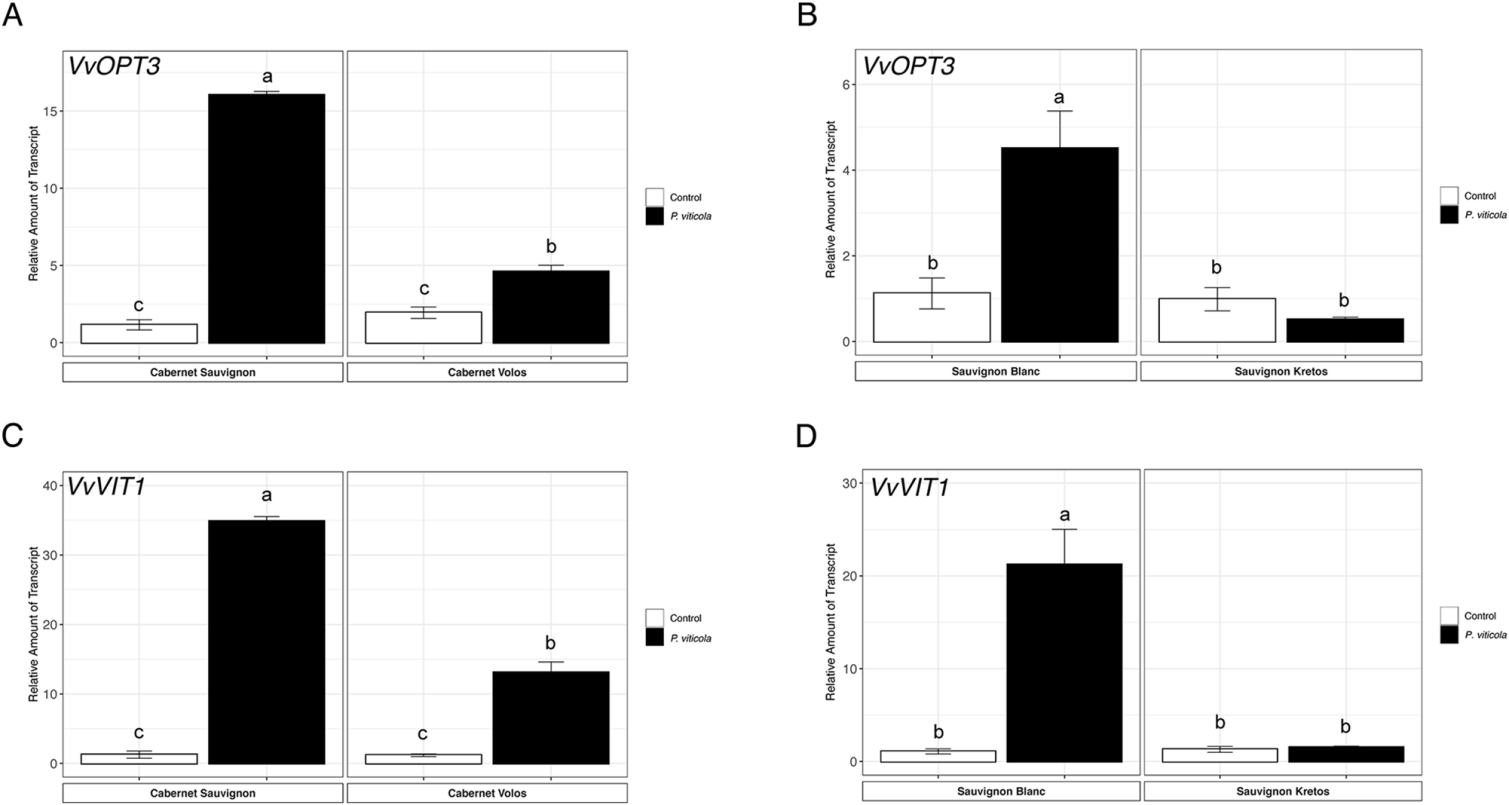
Quantitative RT-PCR analyses of *VvOPT3* and *VvVIT1* genes in grapevine leaves. (**A**) Assessment of *VvOPT3* gene expression in Cabernet Sauvignon and Cabernet Volos, both non-inoculated and inoculated with *Plasmopara viticola*. (**B**) Assessment of *VvOPT3* gene expression in Sauvignon Blanc and Sauvignon Kretos, both non-inoculated and inoculated with *P. viticola*. (**C**) Assessment of *VvVIT1* gene expression in Cabernet Sauvignon and Cabernet Volos, both non-inoculated and inoculated with *P. viticola*. (**D**) Assessment of *VvVIT1* gene expression in Sauvignon Blanc and Sauvignon Kretos, both non-inoculated and inoculated with *P. viticola*. The data were normalised to the *tubulin* and *EF1α* housekeeping gene. The relative expression ratios were calculated using untreated parental genotypes as a calibrator sample. The values reported are means ± SE of three biological replicates. Different letters indicate significant differences according to one-way ANOVA with Tukey post hoc tests (*p* < 0.05).

The expression of *VvYSL* transporters genes is generally higher in inoculated in comparison to the non-inoculated leaves of susceptible cultivars, in both Cabernet Sauvignon and Sauvignon Blanc (Fig. 6). In particular, *VvYSL1a*,* VvYSL1b* and *VvYSL3* were 50-, 114- and 20-fold up-regulated in inoculated as compared to the non-inoculated Cabernet Sauvignon leaves, respectively (Fig. 6A,C,E). Similarly, *VvYSL1a*,* VvYSL1b* and *VvYSL3* were up-regulated by 40-, 7- and 7-folds in inoculated as compared to non-inoculated Sauvignon Blanc leaves, respectively (Fig. 6B,D,F). Concerning the resistant cultivars, only the Cabernet Volos inoculated leaves showed an up-regulation of *VvYSL1b* as compared to the respective non-inoculated sample (Fig. 6C). Consistently, *VvOPT3* and *VvVIT1* were up-regulated by *P. viticola* inoculation in Cabernet Sauvignon and Sauvignon Blanc leaves (Fig. 7). In addition, *VvOPT3* and *VvVIT1* were up-regulated in inoculated as compared to non-inoculated Cabernet Volos leaves (Fig. 7A,C), whilst no modulations were detected in inoculated leaves of Sauvignon Kretos as compared to the respective non-inoculated samples (Fig. 7B,D).

#### Manganese transporters

**Figure 8:**
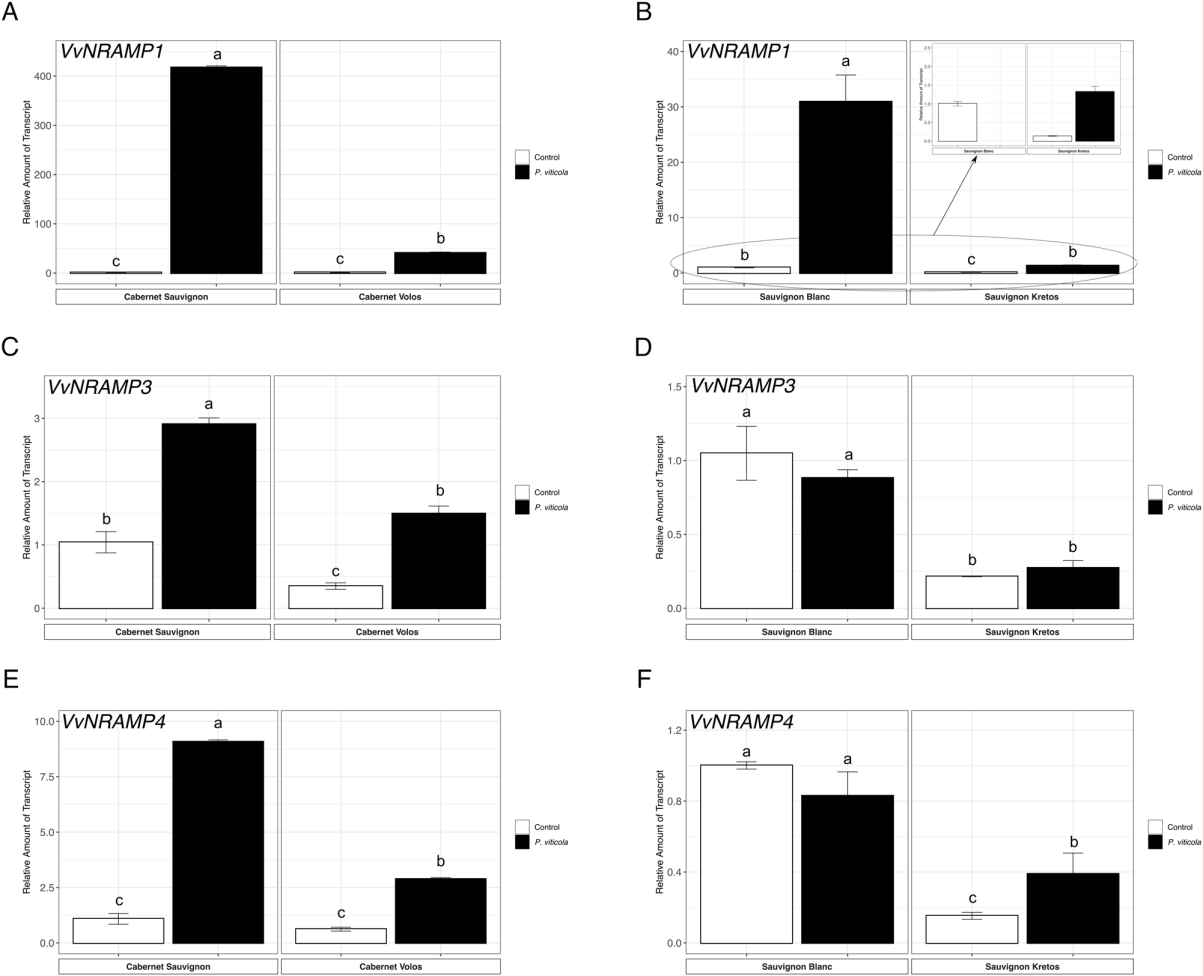
Quantitative RT-PCR analyses of NRAMP (natural resistance-associated macrophage protein) metal-ion transporter genes (*VvNRAMP1*,* VvNRAMP3 and VvNRAMP4*) in grapevine leaves. (**A**) Assessment of *VvNRAMP1* gene expression in Cabernet Sauvignon and Cabernet Volos, both non-inoculated and inoculated with *P. viticola*. (**B**) Assessment of *VvNRAMP1* gene expression in Sauvignon Blanc and Sauvignon Kretos, both non-inoculated and inoculated with *P. viticola*. (**C**) Assessment of *VvNRAMP3* gene expression in Cabernet Sauvignon and Cabernet Volos, both non-inoculated and inoculated with *P. viticola*. (**D**) Assessment of *VvNRAMP3* gene expression in Sauvignon Blanc and Sauvignon Kretos, both non-inoculated and inoculated with *P. viticola*. (**E**) Assessment of *VvNRAMP4* gene expression in Cabernet Sauvignon and Cabernet Volos, both non-inoculated and inoculated with *P. viticola*. (**F)** Assessment of *VvNRAMP4* gene expression in Sauvignon Blanc and Sauvignon Kretos, both non-inoculated and inoculated with *P. viticola*. The data were normalised to the *tubulin* and *EF1α* housekeeping gene. The relative expression ratios were calculated using untreated parental genotypes as a calibrator sample. The values reported are means ± SE of three biological replicates. Different letters indicate significant differences according to one-way ANOVA with Tukey post hoc tests (*p* < 0.05).

The *NRAMP* (Natural Resistance-Associated Macrophage Protein) genes encode primarily for manganese (Mn) transporters, albeit they can also transport other divalent cations (i.e. Fe, Cu and Zn) with a lower specificity^14,59^. According to a previous work, *V. vinifera* genome encodes three putative NRAMP transporters, i.e.* VvNRAMP1*, *VvNRAMP3* and *VvNRAMP4*^14^. The expression of *VvNRAMP* genes were up-regulted in inoculated as compared to non-inoculated leaves of both Cabernet Sauvignon and Cabernet Volos (Fig. 8A,C,E). Concerning the white cultivars, *VvNRAMP1* was up-regulated in both Sauvignon Blanc and Sauvignon Kretos by *P. viticola* inoculation as compared to the respective non-inoculated samples (Fig. 8B). In addition, the inoculated leaves of Sauvignon Kretos showed the up-regulation of *VvNRAPM4* as compared to the non-inoculated leaves (Fig. 8F). On the other hand, *VvNRAMP3* was not modulated in both Sauvignon Blanc and Sauvignon Kretos (Fig. 8D).

## Discussion

The need to find sustainable alternative approaches to control *P. viticola* on grapevine requires to deepen our knowledge on the *Rpv* genes. Since the identification of *Rpv* genes, which have been associated with different phenotypic traits of downy mildew resistance in several grapevine species, breeding programs were undertaken in order to introduce resistant traits in susceptible grapevine species^29,31–33,44,60,61^. Because very little is known on the interaction between elements in leaf tissues and resistance to diseases, the present research aims at clarifying the role played by the ionomic signature of the leaf tissue in the response to the inoculation with *P. viticola* in susceptible cultivars, namely Cabernet Sauvignon and Sauvignon Blanc, in comparison to the respective resistant genotypes carrying the *Rpv12* gene (Cabernet Volos and Sauvignon Kretos). With respect to this resistance trait, *Rpv12* can activate an effective hypersensitive response within 24–48 h post-inoculation with the pathogen, thus preventing the sporulation of *P. viticola*^33^. However, recent pieces of evidence obtained in other species (e.g. *Lactuca sativa* and *Olea europea*) highlighted that also the mineral element balance of plants might play an important role in the resistance mechanisms against pathogens^52,53^. Although the compatible and the incompatible interaction between *V. vinifera* plants and *P. viticola* have been thoroughly characterized at transcriptomic, proteomic and metabolomic levels^21–26^, to the best of our knowledge, no information on the modulation of the ionomic profile in infected leaves of both susceptible and resistant cultivars is available. The analysis of the ionomic signature of *V. vinifera* leaves non-inoculated and inoculated with *P. viticola* highlighted a different concentration and/or distribution of several mineral nutrients in the leaf. In particular, in susceptible cultivars, in addition to their differential distribution in the leaves upon the infection with *P. viticola*, there is also an increase in specific microelement concentrations (i.e. Mn, Fe, Zn). Such differential allocation in the leaves has been also detected for several macronutrients (i.e. P, S, K and Ca), even though the total leaf concentration of these elements was not affected by the infection. Yet, in resistant cultivars, despite the infection with *P. viticola* could enhance the total leaf concentration of microelements (i.e. Mn and Fe), the spatial distribution in the leaf is not altered.

In susceptible cultivars, the ionomic profiling revealed that *P. viticola* infection caused an increase in Fe concentration, whilst the application of µXRF revealed that such increase was uniformly distributed all over the whole leaf. In Cabernet Sauvignon and Sauvignon Blanc, genes putatively related to Fe partitioning and translocation towards the sink tissues^62–67^, such as *VvYSL1a*, *VvYSL1b*, *VvYSL3* and *VvOPT3*, were significantly up-regulated after *P. viticola* inoculation. Consistently, the up-regulation of the *VvVIT1* gene, whose orthologous in other plants are involved in Fe, Mn and Zn trafficking across the tonoplast membrane^68,69^, suggested a re-arrangement in Fe distribution in the inoculated leaves. It has been postulated that the inoculation of plants with specific pathogens might induce a “Fe deficiency-like” signal^70^. Consistently, the silencing of *AtYSL3* was related with an enhanced susceptibility of *A. thaliana* plants toward the pathogen *Pseudomonas syringae* pv. *tomato* DC3000^71^. Although the µXRF did not highlight alterations in Fe distribution maps, the leaf micronutrient concentration was increased by *P. viticola* infection only in Sauvignon Kretos plants. Unexpectedly, genes putatively involved in Fe partitioning (i.e.* VvYSL1a*, *VvYSL1b*, *VvYSL3*,* VvOPT3* and *VvVTI1*) were not modulated in inoculated Sauvignon Kretos leaves, whilst transporters belonging to the NRAMP family, (*VvNRAMP1* and *VvNRAMP4*), were up-regulated by *P. viticola* infection. Interestingly, NRAMP transporters have been shown to be both involved in the Fe starvation response, in the uptake and translocation of divalent cations, like Mn, Fe, Cu and Zn^14,59,72–74^ and to be induced by infections in *A. thaliana* plants^75^. Inoculated Cabernet Volos leaves showed an up-regulation *VvYSL1b*, *VvOPT3* and *VvVTI1* that suggested a redistribution of Fe in the leaf, even though the total concentration was not affected. A possible involvement of Fe in the resistance mechanisms against pathogens has been for long time postulated; for instance, at rhizosphere level, the competition for Fe between pathogens and beneficial microbes (i.e. plant growth-promoting rhizobacteria) can limit the growth of dangerous microorganisms, thus indirectly protecting plants from diseases^76,77^. However, there is also an increasing body of evidence suggesting that the inoculation with pathogens can lead to the disturbance of Fe homeostasis^78–81^. The local increase in Fe concentration has been hypothesised to be associated with the production of Reactive Oxygen Species (ROS), which are well known to be involved in the hypersensitive response^82^. Moreover, this resistance trait has been identified as distinctive for the grapevine resistant cultivars carrying the *Rpv12* gene^33^. Even though free Fe can catalyse ROS formation^83^, up to date its role in the ROS production in response to pathogen inoculation is still under investigation^70^. Considering the possible role of this micronutrient in the hypersensitive response^82^, the difference in Fe concentration and the differential modulation of genes putatively involved in Fe translocation and partitioning in the leaves of resistant cultivars inoculated with the pathogen might be ascribable to a different timing in the response of plants toward the pathogen. In fact, at the sampling time, Sauvignon Kretos seemed to have already translocated Fe to the leaves (i.e. high Fe concentration in the leaves and reduced expression of genes involved in partitioning) suggesting a faster reaction to the *Plasmopara*. On the other hand, inoculated leaves of Cabernet Volos featured a Fe concentration similar to non-inoculated ones, whilst the genes involved in Fe partitioning were up-regulated, thus possibly suggesting an ongoing Fe translocation process at the time of sampling.

In Cabernet Sauvignon and Sauvignon Blanc cultivars, the inoculation with *P. viticola* increases Mn concentration in the leaf. In particular, the micronutrient was strongly accumulated on the necrotic lesion induced by the pathogen, with the mineral element mainly localised at the borders of infected spots. Such increase in Mn concentration in the infected leaves of susceptible cultivars might be associated to the enhanced expression of *VvNRAMP* genes, which could be putatively involved in the uptake and translocation of this cation^84,85^. Manganese is indeed accounted as one of the most effective micro-elements in the suppression of plant diseases^86^, being able to affect several biochemical reactions involved in the synthesis of phenolic compounds (e.g. flavonoids) and of lignin and suberin, with some of them showing antimicrobial activities^56,87^. In cucumber plants infected with pathogenic fungi, Mn was shown to reduce the sensitivity by regulating the synthesis of the cell wall polysaccharides and the cell water status^88^. These pieces of evidence might therefore suggest an active process of the cell wall strengthening in the lesions, in particular at the boundaries of the *P. viticola* inoculation spots. Similar dynamics for Mn were also observed in resistant cultivars, which showed both a higher Mn concentration and an increased expression of *VvNRAMP* genes in the infected leaves as compared to non-infected ones. Noteworthy, both the concentration of Mn and the expression levels achieved in the infected leaves of resistant cultivars were significantly lower as compared to the infected leaves of the respective susceptible cultivars.

In spite of being primarily characterised as Mn transporters in plants^84,85^, members of the *NRAMP* gene family have been also shown to mediate the uptake and translocation of other divalent cations, like Fe, Cu and Zn, albeit less specifically^14,59^. Therefore, the up-regulation *VvNRAMP* genes in the susceptible cultivars infected with *P. viticola* might be also related to the modification in the distribution of Zn, which has been suggested to have a role in plant cell protection against the oxidative stress and in the production of important disease resistance signalling proteins^56,89^. Consistently with the dynamics observed in the susceptible cultivars, the up-regulation of *VvNRAMP* genes in the inoculated resistant cultivars did not cause a differential accumulation of Zn in the leaves as compared to the respective non-inoculated controls.

The inoculation of susceptible cultivars with *P. viticola* also modified the distribution of S, even though the total leaf concentration did not change as compared to the non-inoculated plants. Indeed, the modulation of transporter genes might suggest a redistribution of S at cellular/subcellular levels, whereas the steady S concentration in the tissues might be accounted to the presence of S in the organic form. On the other hand, the S distribution was not affected by *P. viticola* inoculation in resistant cultivars (i.e. Cabernet Volos and Sauvignon Kretos). However, the inoculation up-regulated *VvSULTR3.5* in leaves of Cabernet Sauvignon, Cabernet Volos and Sauvignon Blanc, albeit to significantly lower levels in the resistant cultivar. Interestingly, the ortholog gene *PtSULTR3.5* was up-regulated in poplar leaves inoculated with the fungus *Melampsora larici-populina*^90^, further strengthening the connection between S dynamics in leaves and plants response to biotic stresses. Indeed, S is involved in different metabolic pathways in plant cells, such as the synthesis of hydrogen sulphide, cysteine, methionine, glutathione, glucosinolates and phytoalexins, to tackle with biotic stressors^91,92^.

The distribution of other macroelements, like K, Ca and P, resulted modified only in the inoculated leaves of susceptible varieties, even though their possible roles in the response to pathogen are still under investigation. An adequate K nutrition in plants can contribute in reducing the susceptibility towards bacterial and fungal infections^56,93^. At cell level, K fluxes are involved in the control on the plasma membrane polarization^93^; whose alterations are often observed in the presence of fugal effectors^94^. Changes in the membrane polarity quickly occur after the infection and they trigger intracellular signalling cascades, involving, for instance, Ca as second messenger^95^. Besides being involved in transient intracellular oscillation activating signalling cascades, Ca can also play a structural role in cells, stabilizing and strengthening the cell wall^56^; this would explain the increased Ca concentration in the *P. viticola*-induced lesion. In addition, P also showed an accumulation at the infection lesions; although P plays a paramount role in plants life cycle, its involvement in the resistance mechanisms against pathogens is still controversial, due to contrasting observations^86^. Nonetheless, it has been observed that the foliar application of orthophosphoric acid, or its salts, can inhibit the diseases caused by Oomycota belonging to the *Phytophthora* and *Pythium* species^96^.

In conclusion, by investigating the compatible and the incompatible interaction between *V. vinifera* plant and *P. viticola*, we demonstrated that in susceptible cultivars, the inoculation with *P. viticola* caused a dramatic redistribution of several micro- and macronutrients (e.g. Fe, Mn, Zn, S, K, Ca and P), in terms of concentration and localization at leaf level. This evidence might therefore suggest a role of Fe, Mn, Zn, S, K, Ca and P in the response to the pathogen, which could be exerted according with the specific functions of single elements in the plant cell during the compatible interaction. Such differential accumulation and allocation of elements was associated to the modulation of specific transporters by *P. viticola* inoculation (i.e.* VvYSL1a*,* VvYSL1b*,* VvYSL3*,* VvOPT3*,* VvVIT1*,* VvNRAMP1*,* VvNRAMP3*,* VvNRAMP4* and *VvSULTR3.5*). On the contrary, the resistant cultivars did not display a substantial alteration in the element distribution in the leaves infected with *P. viticola*. However, Mn and Fe showed an increased concentration in inoculated leaves that is also mirrored by the transcriptional regulation of putative transporters, suggesting that Cabernet Volos and Sauvignon Kretos included some pathogen defence mechanisms based on the increase of Mn ad Fe concentration. These two elements are involved in the synthesis of secondary metabolites (e.g. phenolic compounds)^56,87^ and ROS^70,82,83^, which play a key role in the hypersensitive response observed in incompatible interactions. Our results clearly indicate the link between the mineral nutrition (in terms of nutrient contents and localisation) and plants’ response to pathogens. This evidence suggests that an appropriate management of the mineral element availability for grapevine plants at field scale could play a pivotal role in guaranteeing the expression of the response mechanisms against pathogens. In fact, a mineral element shortage, even though latent or induced by toxicity/excess of other mineral elements, as already demonstrated for Cu^14^, can seriously limit the ability of either susceptible or resistant grapevine plants to cope with *P. viticola* infections.

## Materials and methods

### Plant material, growing conditions and *Plasmopara viticola* inoculation

Tissues were sampled from one-year old grapevine (*V. vinifera*) cuttings grafted to SO4 rootstock plants, belonging to four different cultivars, two resistant (Sauvignon Kretos and Cabernet Volos) and two susceptible (Sauvignon Blanc and Cabernet Sauvignon). The resistant cultivars were obtained through two crosses between *V. vinifera* cv Sauvignon Blanc or Cabernet Sauvignon with the *Vitis* ‘breeding line 20/3’, obtained by Pal Kozma in Ungary and kindly offered to the University of Udine. The crosses were performed at the University of Udine (Udine, Italy). The *Vitis* 20/3 has been obtained from a crossing between *Vitis* ‘Bianca’ x ‘SK77-4/5’ (bred by crossing ‘Kumbarát’, originated from hybridisation of *V. amurensis *x *V. vinifera*, and *V. vinifera* ‘Traminer’)^97–100^. The two susceptible cultivars (Sauvignon Blanc and Cabernet Sauvignon) were used as control. All the plants were obtained from Vivai Cooperativi Rauscedo (Rauscedo, Italy).

Plants were grown in 4.2 L pots (12 × 12 × 30 cm) where at the bottom was placed a layer of expanded clay. Before the planting, the peat soil of each pot was supplemented with the urea fertilizer (to a concentration of 20 kg N Ha^−1^) and manually mixed. The roots of each grapevine were cut to approximately 10 cm length. The pots were manually irrigated, every 2–3 days, in order to maintain constant soil humidity during the whole experiment. Plants were grown in an experimental greenhouse under controlled relative humidity (RH) and temperature conditions (65% ± 5% RH, 24 °C ± 1 °C).

Grapevine plants were inoculated by spraying the abaxial side of the leaves with a solution containing 5 × 10^5^ spores of *P. viticola* as previously described^101^. Inoculated and non-inoculated, were kept at 100% RH overnight to allow pathogen infection and then incubated under greenhouse conditions. Six days after the inoculation the RH of the chambers was again increased up to 100%, in order to allow the pathogen sporulation. The day after, leaves were sampled, immediately frozen in liquid N_2_ and stored at -80 °C until further processing. For qPCR and mineral analysis, each sample (biological replicate) comprised three leaves taken from the same plant and only leaves of the 4th–5th node from the top of the shoot were collected to avoid ontogenic resistance effects^101^. Three independent biological replicates were analysed; each biological replicate was analysed three times, as for technical replicates.

### Ionomic analysis

The determination of leaves ionomic signature was carried out as previously described^102^. Briefly, samples of leaves tissue were digested with concentrated HNO_3_ [65% (v/v), Carlo Erba] using a single reaction chamber (SRC, UltraWAVE, Milestone Inc, Shelton, USA). The mineral elements concentration was subsequently determined by Inductively Coupled Plasma-Optical Emission Spectroscopy (ICP-OES; Arcos Ametek, Spectro, Germany), using tomato leaves (SRM 1573a) and spinach leaves (SRM 1547) as external certified reference material.

### Micro-focused X-ray fluorescence (μ-XRF) imaging

Micro X-ray fluorescence maps were collected with a laboratory benchtop μ-XRF spectrometer (M4 Tornado, Bruker Nano GmbH, Berlin, Germany) at the “Micro X-ray Lab” of the University of Bari, Italy, following the procedure previously reported^103^. This instrument is equipped with a micro-focus Rh X-ray source (50 kV, 600 μA), a polycapillary X-ray optics with a spotsize of 25 μm and two XFlash energy dispersive silicon drift detectors with 30 mm^2^ sensitive area and an energy resolution of 140 eV @ Mn Kα. The two detectors, placed at opposite sites compared to the X-ray optics, allow reducing shadowing effects in the mineral element distribution maps and obtaining a better signal-to-noise ratio (S/N). Immediately after sampling, grapevine leaves were inserted between two circles of filter paper and kept tightly pressed within a Petri dish to preserve the sample flat during the freeze-drying process; the Petri dishes containing the flat leaves were quickly frozen in liquid nitrogen and then freeze-dried under vacuum. All the µ-XRF analyses were performed under reduced pressure (20 mbar) by using 25 μm stepsize and an acquisition time of 10 ms per step. In order to increase the S/N, each scan was repeated 30 times. For each of the leaves, a rectangular area of 220–240 pixels (ca. 5.5–6 mm) height and 400–440 pixels (ca. 10–11 mm) width (depending on leaf dimension), was selected approximately near the petiole sinus (including the midrib and nearby primary vein) of the leaf for the analysis. Smaller areas (approximately 1 mm height and 1.5 mm width) containing infection lesions were also analysed keeping the acquisition conditions unchanged, except for the use of a smaller step size (10 µm) to improve map details through oversampling. µ-XRF distribution maps were obtained with the instrument ESPRIT software (Bruker Nano GmbH, Berlin, Germany) version 1.3.0.3273. All the mineral element distribution maps were collected using the same analytical conditions. The same intensity scale was adopted to visualise the distributions of the same mineral element in all the maps. Therefore, the mineral element distribution maps of the same mineral element can be directly compared. Brighter colours in the map correspond to a higher concentration of the mineral element. Several areas on many different leaves were investigated and the reported data can be considered representative of the main and more relevant features observed in all the studied samples.

### Bioinformatics

The identification of transporter gene sequences, namely *VIT1*, *OPT3*, *YSL1*, *SULTR3*, involved in the translocation and allocation of selected mineral elements was carried out in the 12X release of *Vitis vinifera* genome in the Grape Genome Browser (https://www.cns.fr/externe/GenomeBrowser/Vitis/). The isolation of putative gene sequences from the grapevine genome has been primarily based on amino acid sequence similarity between already characterised transporters (Supplementary Table 1), retrieved from public databases like https://www.ncbi.nlm.nih.gov/, https://www.uniprot.org/uniprot/. The predicted transporter sequences were identified in grapevine genome through a BLASTP^104^ search. BLASTP analysis was performed using each known protein, selecting the putative proteins encoded by the predicted coding sequences on the basis of the highest sequence homology value (the threshold value for sequence homology was set at 80%); afterwards a phylogenetic analysis was performed. The amino acid sequences were aligned by the ClustalW ver. 2.1 algorithm (https://clustalw.ddbj.nig.ac.jp/). Phylogenetic tree was built using the Phylogenetic Interference Package program (PHYLIP; University of Washington, USA, https://evolution.genetics.washington.edu/phylip.html) and visualised by the Figtree software (https://tree.bio.ed.ac.uk/software/figtree/).

#### Sulphate transporters

On the basis of the predicted functions, the bioinformatic analysis was aimed at identifying specifically members of the group 3, by using orthologous sequences from *A. thaliana* and *Oryza sativa* (Supplementary Table 1). The BLASTP algorithm identified eight putative transporters in the genome of *V. vinifera*; one sequence (*GSVIVT01022159001*) resulted more similar to group 2 transporters (Supplementary Figure 2), whereas seven sequences (*GSVIVT01001198001*,* GSVIVT01015413001*,* GSVIVT01018027001*,* GSVIVT01015414001*,* GSVIVT01018028001*,* GSVIVT01011744001*,* GSVIVT01015412001*) clustered with other transporter sequences belonging to the group 3. Among the putative group 3 transporters, five sequences (*GSVIVT01018027001*, *GSVIVT01018028001*,* GSVIVT01011744001*,* GSVIVT01015412001* and *GSVIVT01015413001*, hereafter referred to as *VvSultr3.1*,* VvSultr3.2*,* VvSultr3.3*,* VvSultr3.4*,* VvSultr3.5*) were kept for the gene expression analyses, whereas *GSVIVT01015414001*and *GSVIVT01001198001* were discarded since a duplication and a truncated form of *VvSultr3.5* and *VvSultr3.1*, respectively (Supplementary Figure 2).

#### Iron transporters

Members of two distinct clades of the Oligopeptide Transporter family, the Yellow Stripe-like (YSL) proteins and the Oligopetide Transporters (OPTs), have been shown to be involved in Fe partitioning in plants^62–64,105^. Known YSL protein sequences from different plants, as for instance *A. thaliana*, *Oryza sativa*, *Hordeum vulgare*, *Zea mays* and *Arachis hypogea* (Supplementary Table 1), were used to retrieve six sequences (i.e.* GSVIVT01019645001*, *GSVIVT01019646001*, *GSVIVT01038611001*, *GSVIVT01029329001*, *GSVIVT01029331001*, *GSVIVT01012033001*) in the *V. vinifera* genome. According to the phylogenetic analysis (Supplementary Figure 3), the six sequences isolated in the grapevine genome clustered in two different branches of the phylogenetic tree; *GSVIVT01029329001*, *GSVIVT01029331001*, *GSVIVT01012033001* clustered with AtYSL5, AtYSL7 and AtYSL8, whereas *GSVIVT01019645001* (hereafter referred to as *VvYSL1a*), *GSVIVT01019646001* (hereafter referred to as *VvYSL1b*), *GSVIVT01038611001* (hereafter referred to as *VvYSL3*) showed a closer relationship with the *A. thaliana* sequences (AtYSL1, AtYSL2 and AtYSL3), known to be involved in the Fe partitioning in plants^65–67^.

The already characterised OPT3 sequences from *A. thaliana* and *Populus trichocarpa* (Supplementary Table 1) allowed the identification of five putative OPT sequences in grapevine genome, *GSVIVT01014721001*, *GSVIVT01014724001*, *GSVIVT01009222001*, *GSVIVT01005133001*, *GSVIVT01038599001*, already annotated as *VvOPT1*, *VvOPT1a*, *VvOPT2*, *VvOPT3* and *VvOPT7* respectively. The phylogenetic analysis confirmed that VvOPT3 had the highest homology with the OPT3 sequences from *A. thaliana* and *P. trichocarpa* (Supplementary Figure 4), thus suggesting a similar function in plants.

Two putative *V. vinifera* sequences (i.e. *GSVIVT01011629001* and *GSVIVT01011628001*) showed a high homology with AtVIT1, OsVIT1, OsVIT2 and PtVIT1 from poplar (Supplementary Table 1). According to the phylogenetic analysis (Supplementary Figure 5), *GSVIVT01011629001*, hereafter referred to as *VvVIT1*, was the sequence most closely related to *AtVIT1*; on the bases of this similarity, *VvVIT1* was selected for further molecular analyses.

### RNA extraction and cDNA synthesis

RNA samples were extracted from leaf tissues sampled as described above. Total RNA was prepared using Spectrum Plant Total RNA Kit (Sigma-Aldrich Co. LLC) according to the users’ guide, as previously described^106^. Afterwards, 1 μg of total RNA was subjected to DNAse digestion with 10 U of DNAse RQ1 and cDNA was synthesised using the ImProm-II Reverse Transcription System (Promega, Madison, WI, USA). The quality of total RNA and cDNA was checked through a PCR using couples of primers specific for housekeeping genes.

### Real-time reverse transcription–PCR

Specific primers were designed for the target genes as well as for the housekeeping genes (Supplemental Table 2). Real-time reverse transcription–PCR (RT-PCR) experiments were carried out in biological triplicates and the reaction was performed by using the SsoFast EvaGreen Supermix (Bio-Rad, Segrate, Italy) as previously described^106^. Nevertheless, the identity of each amplicon was confirmed by sequencing. The amplification efficiency was calculated from raw data using LinRegPCR software^107^. The expression data were normalized to the *tubulin* and *EF1α* housekeeping genes, whereas the relative expression ratios were calculated using non-inoculated susceptible cultivar as a calibrator sample according to the Pfaffl equation^108^. Standard error values were calculated according to Pfaffl et al. (2002).

### Statistical analyses

The results are reported as mean ± standard error (SE). The significance of differences among means was calculated by One-way ANOVA with post-hoc Tukey HSD with α = 0.05 using R software (version 3.6.0). The following R packages were used for data visualization and for statistical analyses: ggplot2 v.3.2.0^110^, Agricolae v.1.3-1^111^ and ggfortify^112^.

## Supplementary information

Supplementary information.
