# Supplementary material for: Plasmopara viticola infection affects mineral elements allocation and distribution in Vitis vinifera leaves
Source: Sci Rep. 2020 Oct 30;10:18759. doi: 10.1038/s41598-020-75990-x (PMC7603344; doi:10.1038/s41598-020-75990-x)
Supplement: Supplementary file 1 — Supplementary information. [file 41598_2020_75990_MOESM1_ESM.pdf]

***Plasmopara viticola* infection affects mineral elements allocation and distribution in *Vitis vinifera* leaves.**

Stefano Cesco[1], Anna Tolotti[1], Stefano Nadalini[2], Stefano Rizzi[2], Fabio Valentinuzzi[1], Tanja Mimmo[1], Carlo Porfido[3], Ignazio Allegretta[3], Oscar Giovannini[4], Michele Perazzolli[4,5], Guido Cipriani[2], Roberto Terzano[3], Ilaria Pertot[4,5], Youry Pii[1,*]

[1]Faculty of Science and Technology, Free University of Bolzano-Bozen, I-39100 Bolzano, Italy
[2]Department of Agricultural, Food, Environmental and Animal Sciences, University of Udine, I-33100 Udine, Italy.
[3]Department of Soil, Plant and Food Sciences, University of Bari “Aldo Moro’’, Bari, I-70126, Italy
[4]Department of Sustainable Agro-ecosystems and Bioresources, Research and Innovation Centre, Fondazione Edmund Mach, I-38010 San Michele all’Adige, Italy.
[5]Center Agriculture Food Environment (C3A), University of Trento, I-38010 San Michele all’Adige, Italy.

*Corresponding author:

Youry Pii

Faculty of Science and Technology, Free University of Bozen-Bolzano

Piazza Università 5, I-39100

Bolzano, Italy

Phone: +390471017164

e-mail: youry.pii@unibz.it

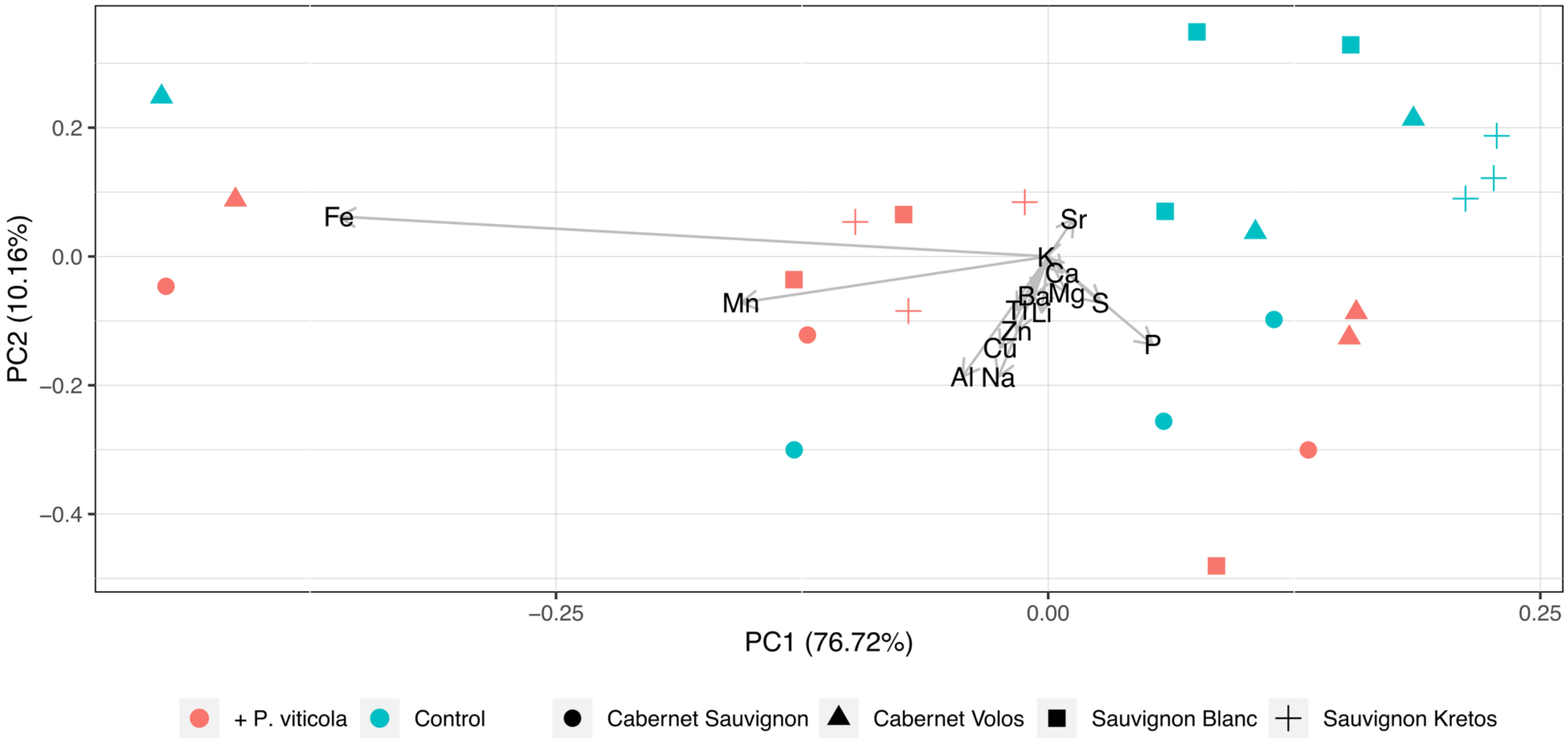

Supplementary Figure 1. Principal Component Analyses of whole ionomic dataset.

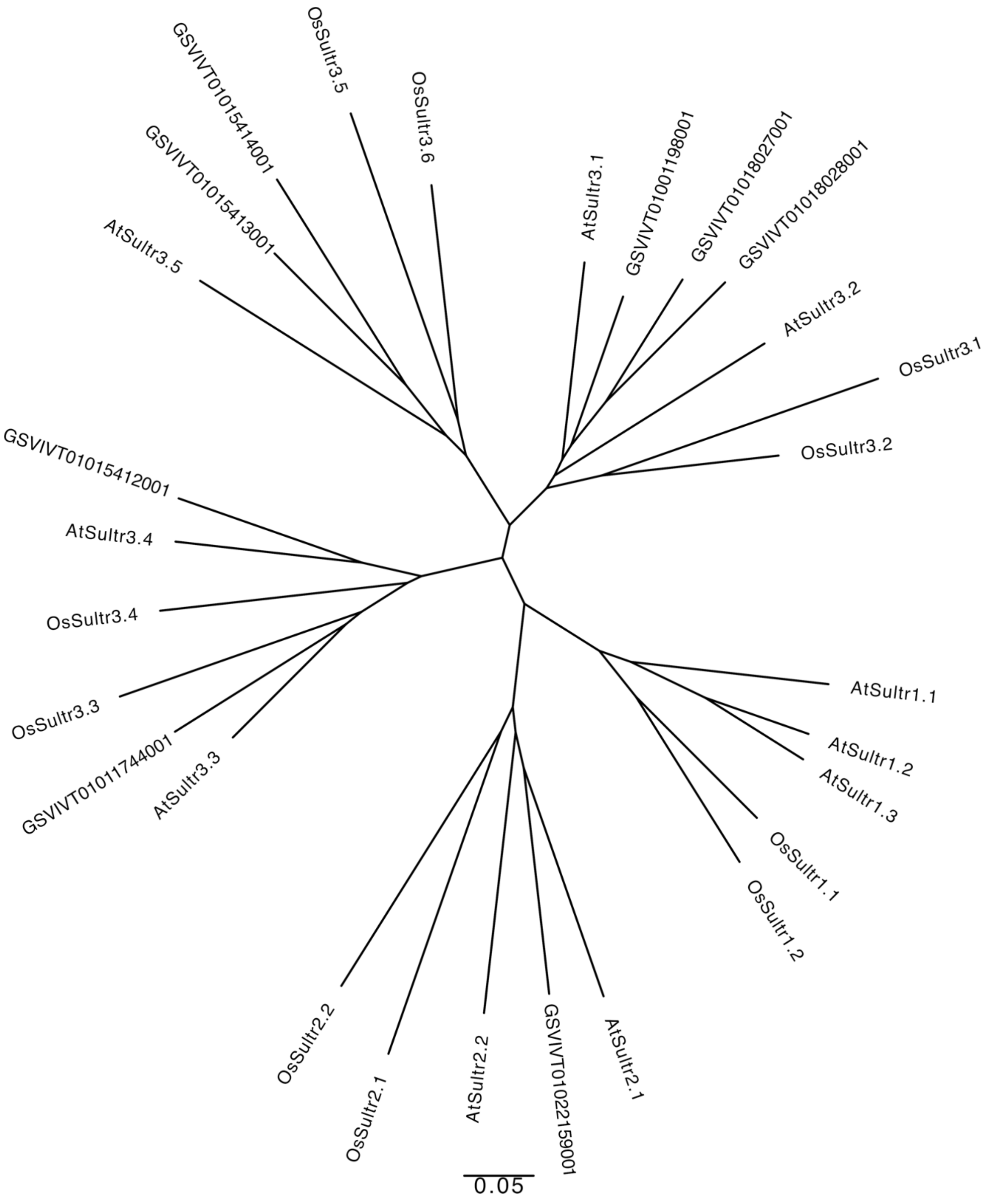

Supplementary Figure 2. Phylogenetic tree showing the relationships among the SULTR3 transporters of *Vitis vinifera*, *Arabidopsis thaliana*, and *Oryza sativa*. Phylogenetic tree was built using the Phylogenetic Interference Package program (PHYLIP; University of Washington, http://evolution.genetics.washington.edu/phylip.html) and visualised by the Figtree software (http://tree.bio.ed.ac.uk/software/figtree/) (for protein ID codes, see Supplementary Table 1). Bootstrap values from 1000 replicates were used to estimate the confidence limits of the nodes. The scale bar represents a 0.05 estimated amino acid substitution per residue.

.

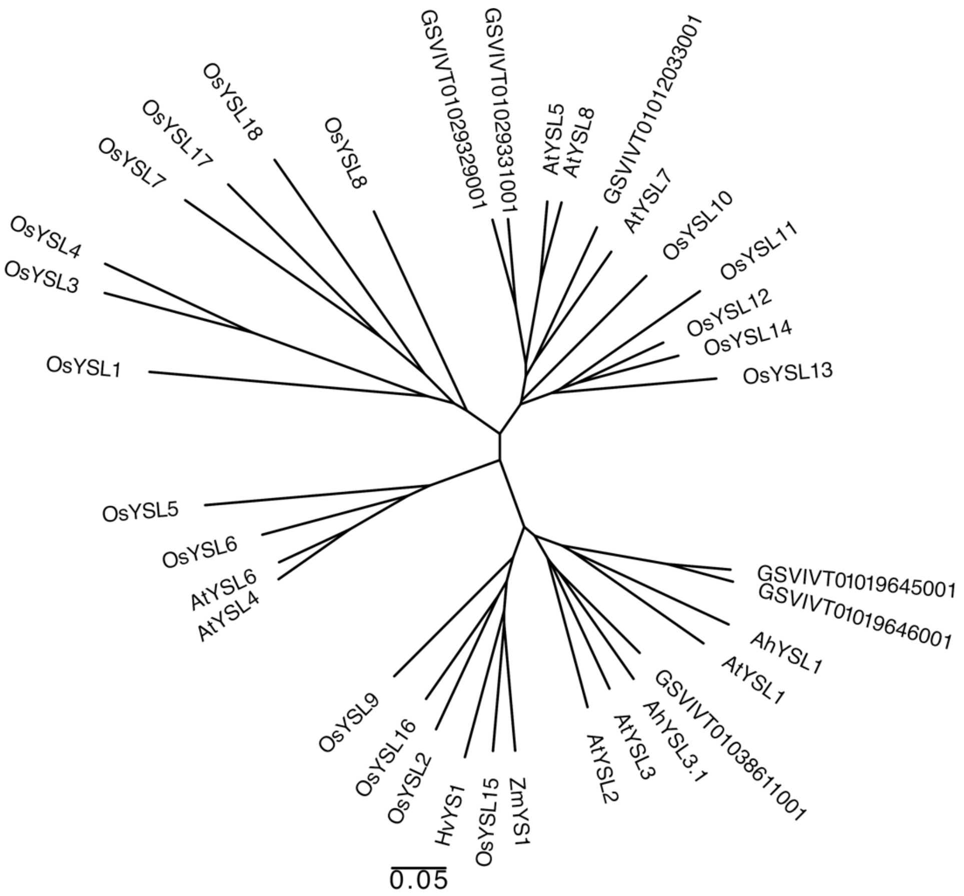

Supplementary Figure 3. Phylogenetic tree showing the relationships among the YSL transporters of *Vitis vinifera*, *Arabidopsis thaliana*, *Oryza sativa*, *Hordeum vulgare*, *Zea mays* and *Arachis hypogea*. Phylogenetic tree was built using the Phylogenetic Interference Package program (PHYLIP; University of Washington, http://evolution.genetics.washington.edu/phylip.html) and visualised by the Figtree software (http://tree.bio.ed.ac.uk/software/figtree/) (for protein ID codes, see Supplementary Table 1). Bootstrap values from 1000 replicates were used to estimate the confidence limits of the nodes. The scale bar represents a 0.05 estimated amino acid substitution per residue.

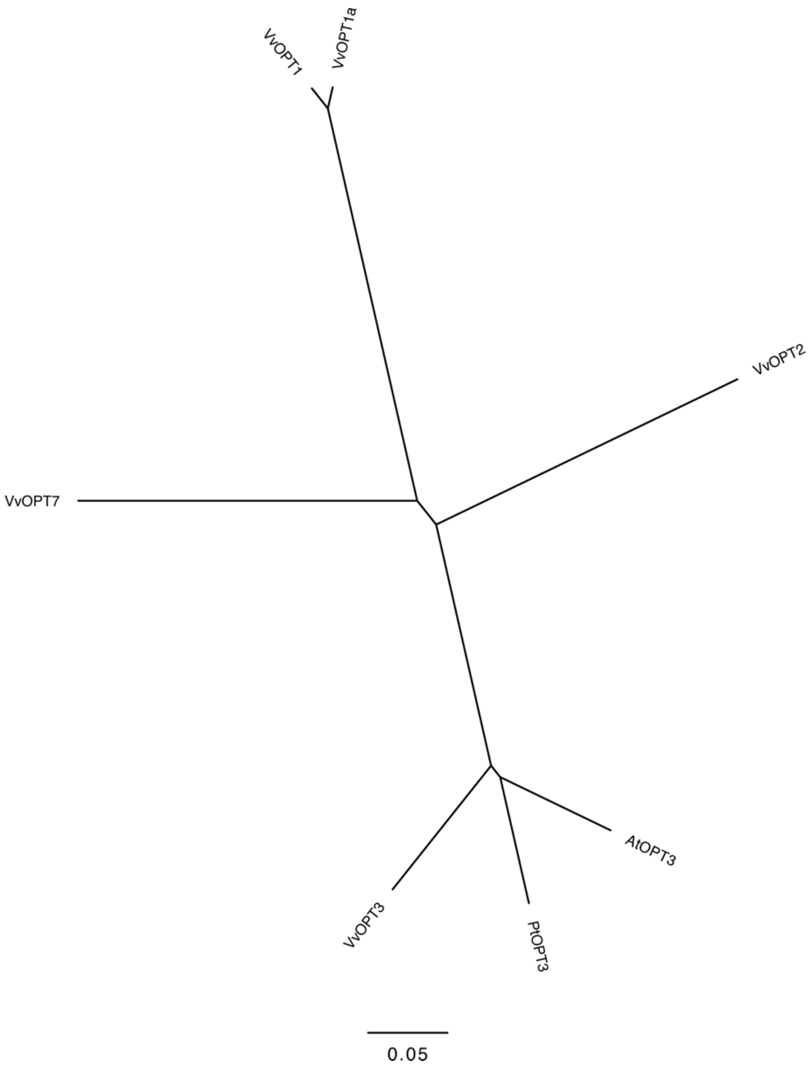

Supplementary Figure 4. Phylogenetic tree showing the relationships among the OPT transporters of *Vitis vinifera*, *Arabidopsis thaliana* and *Populus trichocarpa*. Phylogenetic tree was built using the Phylogenetic Interference Package program (PHYLIP; University of Washington, http://evolution.genetics.washington.edu/phylip.html) and visualised by the Figtree software (http://tree.bio.ed.ac.uk/software/figtree/) (for protein ID codes, see SupplementaryTable 1). Bootstrap values from 1000 replicates were used to estimate the confidence limits of the nodes. The scale bar represents a 0.05 estimated amino acid substitution per residue.

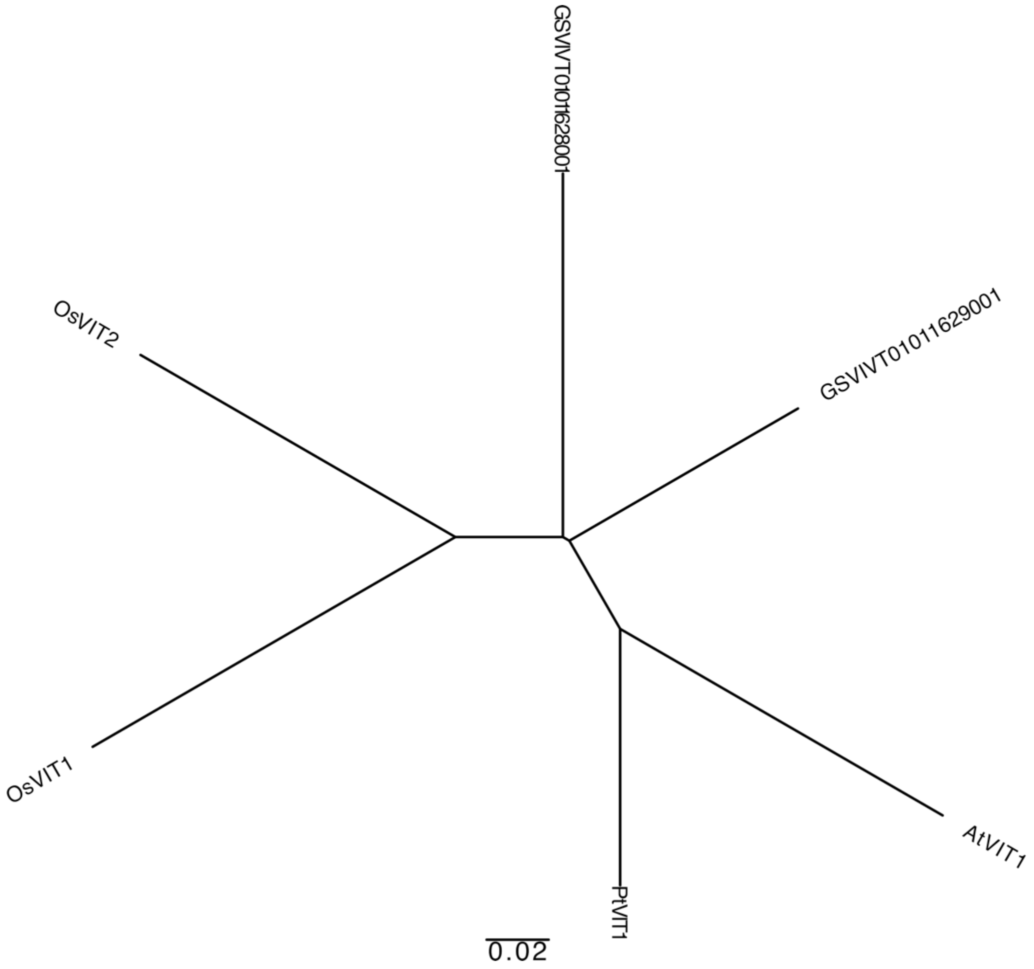

Supplementary Figure 5. Phylogenetic tree showing the relationships among the VIT1 transporters of *Vitis vinifera*, *Arabidopsis thaliana*, *Populus trichocarpa*, and *Oryza sativa*. Phylogenetic tree was built using the Phylogenetic Interference Package program (PHYLIP; University of Washington, http://evolution.genetics.washington.edu/phylip.html) and visualised by the Figtree software (http://tree.bio.ed.ac.uk/software/figtree/) (for protein ID codes, see Supplementary Table 1). Bootstrap values from 1000 replicates were used to estimate the confidence limits of the nodes. The scale bar represents a 0.02 estimated amino acid substitution per residue.

Supplementary Table 1. Nomenclature and accession number of *VIT1*, *YSL*, *OPT3* and *SULTR* genes used for the bioinformatic research of orthologous genes in *V. vinifera* genome and for the generation of phylogenetic trees.

Gene	Protein	Accession Number	Organism
VIT1			
	AtVIT1	ABI93896	*Arabidopsis thaliana*
	OsVIT1	Q6MWE5.1	*Oryza sativa*
	OsVIT2	Q6ERE5.2	
	PtVIT1	ABK94901.1	*Populus trichocarpa*
YSL			
	AhYSL1	JQ248576	*Arachis hypogea*
	AhYSL1.3	JQ248577	
	AtYSL1	At4g24120	*A. thaliana*
	AtYSL2	At5g24380	
	AtYSL3	At5g53550	
	AtYSL4	At5g41000	
	AtYSL5	At3g17650	
	AtYSL6	At3g27020	
	AtYSL7	At1g65730	
	AtYSL8	At1g48370	
	ZmYS1	AF186234	*Zea mays*
	HvYS1	AB214183	*Hordeum vulgare*
	OsYSL1	AB190912	*O. sativa*
	OsYSL2	AB126253	
	OsYSL3	AB190913	
	OsYSL4	AB190914	
	OsYSL5	AB190915	
	OsYSL6	AB190916	
	OsYSL7	AB190917	
	OsYSL8	AB190918	
	OsYSL9	AB190919	
	OsYSL10	AB190920	
	OsYSL11	AB190921	
	OsYSL12	AB190922	
	OsYSL13	AB164644	
	OsYSL14	AB164645	
	OsYSL15	AB190923	
	OsYSL16	AB190924	
	OsYSL17	AB190925	
	OsYSL18	AB190926	
OPT3			
	AtOPT3	NP_567493.5	*A. thaliana*
	PtOPT3	XP_002308764.1	*P. trichocarpa*
SULTR			
	AtSultr1.1	At4g08620	*A. thaliana*
	AtSultr1.2	At1g78000	
	AtSultr1.3	At1g22150	
	AtSultr2.1	At5g10180	
	AtSultr2.2	At1g77990	
	AtSultr3.1	At3g51895	
	AtSultr3.2	At4g02700	
	AtSultr3.3	At1g23090	
	AtSultr3.4	At3g15990	
	AtSultr3.5	At5g19600	
	AtSultr4.1	At5g13550	
	AtSultr4.2	At3g12520	
	OsSultr1.1	Os03g0195800	*O. sativa*
	OsSultr1.2	Os03g0196000	
	OsSultr2.1	Os03g0195500	
	OsSultr2.2	Os03g0195300	
	OsSultr3.1	Os10g0420400	
	OsSultr3.2	Os03g0161200	
	OsSultr3.3	Os04g0652400	
	OsSultr3.4	Os06g0143700	
	OsSultr3.5	Os01g0593700	
	OsSultr3.6	Os01g0719300	
	OsSultr4.1	Os09g0240500	

Supplementary Table 2. Sequences of the gene specific primers designed within this work and used for the qRT-PCR analyses.

Gene	Primer Forward 5' → 3'	Primer Reverse 5' → 3'	Reference
VvVIT1	GTAATAAACCTCTCAGAAGTGC	ATGTATACTCGTTAGGGGATTG	This work
VvOPT3	GATTTGGATACAATGTAGAAGAA	CCTCTCTTTCACAAGCATTTC	
GSVIVT01019645001 (VvYSL1a)	AAAGATTCTACACAGATAGACTG	ATTACTCAATGTATGACTTTATCA	
GSVIVT01019646001 (VvYSL1b)	GCTTCTGGTTTGATTTGTGGA	GAGTTTCCTCTATGAAGCCAA	
GSVIVT01038611001 (VvYSL3)	GCGACTTAGAAAAACCAGAGG	TTACTTATTTGAAACTCTGTCAC	
GSVIVT01011744001 (VvSultr3.3)	ACCAGATAGTGTTTACTTGACG	TGATTCCTAGCCAGAACCATTC	
GSVIVT01015412001 (VvSultr3.4)	AAGAGTGTACGATGGAGATTTC	TAAATGGTAGACACTTGGGAC	
GSVIVT01015413001 (VvSultr3.5)	TTCACTCAATGTATGGACACAA	CGATCCTATGTTCCAAAAACAA	
GSVIVT01018028001 (VvSultr3.2)	GCAAGTCCACTTCTGCGGC	TAAACTATTACAACCCTTTATCCTTATT	
GSVIVT01018027001 (VvSultr3.1)	CAGCATCAAATCCAGAGAGTG	TATTCTACAAATACATAATCATTG	
VvNRAMP1	CATTGTGCCCACTATGATAG	AGAGATTGTGCGGCATAACC	1
VvNRAMP2	CTGGGGTATTGGACTATTGG	TTCTTCAGACGGAGATTGAG	
VvNRAMP3	CATTGTGCCCACTATGATAG	GGATCTGAATAGACTGAAGC	
EF1α	GTGCGTCATAGTTTTCTGCC	AAAGAGGACACGACACAACAT	2
Tubulin	CCCCTCTTCCTTTACTATGA	TCTGGCACTATTACAACTGG	

1. Marastoni, L. *et al.* Morphological root responses and molecular regulation of cation transporters are differently affected by copper toxicity and cropping system depending on the grapevine rootstock genotype. *Front. Plant Sci.* **10**, (2019).
2. Pii, Y., Alessandrini, M., Guardini, K., Zamboni, A. & Varanini, Z. Induction of high-affinity NO_3^- uptake in grapevine roots is an active process correlated to the expression of specific members of the *NRT2* and plasma membrane H^+-ATPase gene families. *Funct. Plant Biol.* **41**, 353–365 (2014).
